# Wdr4 promotes cerebellar development and locomotion through Arhgap17-mediated Rac1 activation

**DOI:** 10.1038/s41419-022-05442-z

**Published:** 2023-01-21

**Authors:** Pei-Rung Wu, Shang-Yin Chiang, Robert Midence, Wen-Chao Kao, Chun-Lun Lai, I-Cheng Cheng, Shen-Ju Chou, Chih-Cheng Chen, Chih-Yang Huang, Ruey-Hwa Chen

**Affiliations:** 1 Institute of Biological Chemistry, Academia Sinica, Taipei, 115 Taiwan; 2 Cardiovascular and Mitochondrial Related Disease Research Center, Hualien Tzu Chi Hospital, Buddhist Tzu Chi Medical Foundation, Hualien, 970 Taiwan; 3 Institute of Biomedical Sciences, Academia Sinica, Taipei, 115 Taiwan; 4 Institute of Cellular and Organismic Biology, Academia Sinica, Taipei, 115 Taiwan; 5 Department of Medical Research, China Medical University Hospital, China Medical University, Taichung, 404 Taiwan

**Keywords:** Developmental neurogenesis, Neural progenitors, Movement disorders, Neurodevelopmental disorders, Cell proliferation

## Abstract

Patients with mutations of WDR4, a substrate adaptor of the CUL4 E3 ligase complex, develop cerebellar atrophy and gait phenotypes. However, the underlying mechanisms remain unexplored. Here, we identify a crucial role of Wdr4 in cerebellar development. *Wdr4* deficiency in granule neuron progenitors (GNPs) not only reduces foliation and the sizes of external and internal granular layers but also compromises Purkinje neuron organization and the size of the molecular layer, leading to locomotion defects. Mechanistically, Wdr4 supports the proliferation of GNPs by preventing their cell cycle exit. This effect is mediated by Wdr4-induced ubiquitination and degradation of Arhgap17, thereby activating Rac1 to facilitate cell cycle progression. Disease-associated Wdr4 variants, however, cannot provide GNP cell cycle maintenance. Our study identifies Wdr4 as a previously unappreciated participant in cerebellar development and locomotion, providing potential insights into treatment strategies for diseases with *WDR4* mutations, such as primordial dwarfism and Galloway-Mowat syndrome.

## Introduction

The cerebellum is responsible for the planning, execution, and adjustment of motor behaviors. More recently, it has also been heavily associated with higher cognitive functions such as learning, attention, and emotion [1]. The achievement of these delicate functions requires the coordination of different cerebellar cell types, including Purkinje neurons, Bergmann glia, granule neurons, and interneurons [2]. For this reason, their numbers and organization are meticulously controlled during development [3]. In the mouse cerebellum, progenitors for these cell types originate from two places, the ventricular zone (VZ) of the IV ventricle and the rhombic lip (RL). Progenitors for Purkinje neurons and Bergmann glia are born in the VZ at embryonic day (E) 10.5-E13.5, where they successively migrate toward the pial surface to form the Purkinje layer. On the other hand, RL-derived radial glial cells migrate tangentially to the dorsal surface of the cerebellar anlagen to give rise to granule neuron progenitors (GNPs) in the external granular layer (EGL) at E12.5-E16.5 [2]. The GNPs in the EGL proliferate rapidly at E17.5-postnatal day (P) 14, and their increased number promotes the formation of cerebellar foliation. After exiting the cell cycle, these postmitotic granule neurons migrate radially, along the Bergmann glia, to form the internal granular layer (IGL). Subsequently, the granule neurons project their axons onto the dendrites of Purkinje neurons in the molecular layer (ML). The cerebellar circuits then finally undergo maturation, becoming fully developed at around P56 [3, 4].

At the molecular level, cerebellum development is precisely controlled by extrinsic and intrinsic factors which influence the proliferation, differentiation, and/or migration of various cerebellar cell types. In particular, a combination of sonic hedgehog (Shh), secreted by Purkinje neurons, and transcription factor Atoh1, expressed in GNPs, is required for the massive proliferation of GNPs during development [5], while glycoprotein Reelin secreted by GNPs controls the migration and alignment of Purkinje neurons [6]. Unfortunately, our understanding of the molecular mechanisms governing cerebellar development is far from complete, and it comes at a cost. Unraveling the mechanisms of these cerebellar malformations lies at the crux of developing intervention strategies relevant to clinical settings.

*WDR4* is one of the genes whose mutations are implicated in cerebellar developmental disorders. It encodes a protein with seven WD40 structural motif repeats, and its mRNA is expressed in various tissues, including the kidney, thymus, liver, stomach, lung, testis, and brain [7]. A variety of *WDR4* mutations, including homozygous missense mutations, homozygous splice site mutations, and compound heterozygous mutations have all been identified in patients with neurodevelopmental disorders, including primordial dwarfism [8–10] and Galloway-Mowat syndrome [11]. These patients exhibit neurodevelopmental defects, accompanied with growth retardation, cerebellar atrophy, motor development delay, and gait dysfunction phenotypes. Interestingly, parents of patients carrying a single wild type (WT) *WDR4* allele do not present any phenotype, indicating the recessive nature of this disease-associated allele. Although clinical findings imply a role of WDR4 in brain development, it has not been experimentally validated yet. Even more so, the molecular mechanisms underlying these neurodevelopmental phenotypes are completely unexplored.

In regard to its functions, WDR4 has interactions with numerous different partners. For example, WDR4 is a substrate adaptor of the Cullin 4 (CUL4) E3 ubiquitin ligase complex, which is responsible for recruiting substrates for ubiquitination [12]. WDR4 is also the noncatalytic subunit of *N*^7^-methylguanosine (m^7^G) methyltransferase, binding to the catalytic subunit METTL1 [13, 14]. This complex catalyzes tRNA m^7^G methylation, which stabilizes tRNA to enhance translation efficiency [15–17]. Furthermore, WDR4 binds and regulates DNA repair enzyme Flap Endonuclease 1 (FEN1), maintaining genome stability [18]. Yet for all we know, it remains unclear whether impairment of any of these functions is responsible for the neurodevelopmental disorders observed in the *WDR4*-mutated patients.

Here, we report the crucial role of *Wdr4* in mouse cerebellum development, locomotion and gait. Wdr4 promotes cerebellar GNP proliferation by inhibiting their cell cycle exit in a cell-autonomous manner, and therefore positively regulates the sizes of IGL and ML. In addition, Wdr4 maintains the organization of Purkinje neurons in a non-cell-autonomous manner. The Wdr4-governed GNP proliferation is mediated, at least in part, by the ubiquitination and degradation of Arhgap17 and the elevation of downstream Rac1 activity, signaling which promotes GNP cell cycle maintenance. The disease-associated Wdr4 variants, however, cannot support GNP cell cycle maintenance. Thus, our study establishes a mouse model for *WDR4* mutation-related neurodevelopmental disorders and provides mechanistic insights into these disorders.

## Materials and methods

### Mice

All animal care and procedures were performed according to the guidelines of the Institutional Animal Care and Use Committee, Academia Sinica. Mice were housed at a density of 2–5 adults/cage or 1–3 adults with 1 litter/cage in a specific-pathogen-free animal facility, in which the light-dark cycle was 12:12 hours. All mouse strains were previously reported: *Wdr4*^*flox/flox*^ [12, 18], *Nestin-Cre* [19], *hGFAP-Cre* [20], *Atoh1-Cre* [21], *Rora-Cre* [22], *Sept4-Cre* mice (founder line OX54-CRE, the Gene Expression Nervous System Atlas Project), and *Ai14* [23]. All mice strains were maintained on a C57BL/6 background. Mice of both genders were used in the histological and biochemical experiments, but only males were used in behavioral tests. In experiments that measure the percentage of cells that exited the cell cycle, mice were subcutaneously injected over the cerebellum with EdU (50 mg/kg, #E10187, Invitrogen) at P1, P2, or P3 and were harvested 48 h later. To test the effect of Rac1 activation on GNP proliferation in the cerebellum, mice received one injection with ML099 (20 mg/kg, CID-888706, #15176, Cayman Chemical Company) or DMSO in PBS at P3 for 4 days before harvest.

### Plasmids

Plasmids encoding mouse *Wdr4* (#MC201825, NM_021322) and *Arhgap17* (#MR210303, NM_001122643) were purchased from OriGene Technologies. These cDNA fragments were then subcloned into pRK5 or pLAS5w.PeGFP with a V5 or Flag tag for transient overexpression or packaging lentiviruses carrying Wdr4, respectively. Lentiviruses were generated as previously described [24]. Wdr4 mutants were generated using site-directed mutagenesis with the following primers: Wdr4 D166A: 5′- TTTGTGCTTACTGCAG**C**CCGGGATGAGAAGATC-3′ and 5′-GATCTTCTCATCCCGG**G**CTGCAGTAAGCACAAA-3′, Wdr4 R172Q: 5′-CGGGATGAGAAGATCC**A**GGTCAGCTGGGCTGCT-3′ and 5′-AGCAGCCCAGCTGACC**T**GGATCTTCTCATCCCG-3′. shRNAs against mouse *Wdr4* (Wang et al. [12]) and *Arhgap17* were obtained from the National RNAi Core Facility at Academia Sinica. The target sequences of the shRNAs were *Wdr4* shRNA #1: 5′-CCGCATAGCATCGAGTCTTTC-3′, *Wdr4* shRNA #2: 5′-ATGACAGTAAGCGTCTGATTC-3′, *Arhgap17* shRNA #1: 5′-CCCAGACCAGTGACGTTAATA-3′, *Arhgap17* shRNA #2: 5′-GCCAGAATATGGAGAGAAATA-3′.

### Antibodies and reagents

Primary antibodies used in this study were rabbit anti-Ki67 (1:300, #ab16667, abcam), mouse anti-Calbindin (1:300, #C9848, Sigma), mouse anti-S100β (1:300, # S2532, Sigma), mouse anti-NeuN (1:300, #MAB377, Millipore), mouse anti-Atoh1 (1:50, #AB_10805299, Developmental Studies Hybridoma Bank), rabbit anti-Arhgap17 (1:1000, #ab229221, abcam), mouse anti-Arhgap17 (for detecting Arhgap17 co- immunoprecipitated with endogenous Wdr4, 1:250, #sc-514438, Santa Cruz Biotechnology), mouse anti-Stag2 (1:500, #sc-81852, Santa Cruz Biotechnology), mouse anti-Mark3 (1:500, #05–680, Millipore), mouse anti-Flag (1:5000, #F3165, Sigma), rabbit anti-GAPDH (1:5000, #GTX100118, GeneTex), rabbit anti-Gli2 (1:1000, # 18989-1-AP, Proteintech), rabbit anti-mouse Wdr4 (1:5000 for western blot and 1:250 for staining) [18], rabbit anti-Actin (1:5000, #GTX109639, GeneTex), mouse anti-HA (1:2500, #3724, Cell Signaling), rabbit anti-V5 (1:5000, #AB3792, Millipore), and mouse anti-Rac1 (1:1000 for western blot and 1:150 for staining, #ab33186, abcam). The immunofluorescence secondary antibodies were goat anti-mouse IgG (H + L), Alexa Fluor 647 (1:300, #A21235, Invitrogen), or anti-rabbit IgG (H + L), Alexa Fluor 568 (1:300, #A11001, Invitrogen). The western blotting secondary antibodies were sheep anti-mouse IgG HRP (#NA931, GE Healthcare) or anti-rabbit IgG HRP (#NA934, GE Healthcare). Apoptotic cells were labeled using a TUNEL kit (#12156792910, Roche).

### Cell culture, transfection, and treatments

N2a cells (#CCL-131, American Type Culture Collection) were cultured in high glucose Dulbecco’s modified Eagle’s medium (DMEM), supplemented with 15% fetal calf serum (FCS), 1% non-essential amino acids (#11140-050, Gibco), 2 mM GlutaMax (#35050-061, Gibco), and 100 U/ml penicillin/streptomycin (P/S, #15140122, Gibco). 293FT cells (#R70007, Invitrogen) were maintained in DMEM with 10% FCS and 100 U/ml PS. Transfection of N2a cells was performed using Lipofectamine 3000 reagent (#L3000-015, Invitrogen). For packaging lentiviruses, 293FT cells were transfected using calcium phosphate methods. To block proteasomal degradation, N2a cells were treated with MG132 (1 μM, #474790, Calbiochem) for 16 h. To test protein stability, N2a cells were treated with Cycloheximide (CHX, 100 μg /ml, #01810, Sigma) for different time periods. The cell lines were authenticated by the vendors, and were tested to make sure no mycoplasma contamination in our laboratory routinely.

GNP purification and culture were performed according to a previous study [25]. Briefly, GNPs were purified from P7 mice by Percoll gradient sedimentation. Next, enriched GNPs were pre-plated on a Petri dish to remove glia contamination. At the same time, GNPs were treated with EdU (20 μM) for 2 h and then washed with PBS twice. Purified GNPs were maintained in Neurobasal medium containing B27 supplement, 2 mM GlutaMax, 100 U/mL P/S (all from Invitrogen), 1× SPITE medium supplement, and 1× linoleic acid–oleic acid (all from Sigma), and then plated on dishes coated with poly-D-lysine (10 µg/ml, #P6407, Sigma). For proliferation assays, 1 × 10^6^ GNPs were plated in one well of 12-well dishes in the presence of mouse recombinant Shh (0.5 ng/mL, #464-SH, R&D Systems), cultured for 72 h, and then harvested to count total cell number using Countess II FL Automated Cell Counter (Invitrogen). For immunofluorescence staining and analysis, GNPs were treated with EdU and cultured on coverslips under the same culture conditions, fixed with 4% paraformaldehyde (PFA) for 30 min, and then stained with various antibodies and reagents. To test the effects of ML099, Wdr4 WT or mutants on GNP proliferation, GNPs were isolated, treated with EdU, cultured under the same conditions, treated with ML099 (100 nM) or infected with lentiviruses carrying GFP and Wdr4 WT or mutants at DIV 0, and then harvested 72 h later for proliferation, staining, or western blot analysis.

### Peptide preparation for mass spectrometry analysis

P7 cerebellar GNPs from *Wdr4 A-cKO; Ai14* (tdTomato) and control mice were isolated using FACSAria IIIu (Becton Dickinson, NJ, USA). 5 × 10^5^ GNPs were lysed with 8 M urea in 25 mM ammonium bicarbonate. After lysis and centrifugation, the extracted proteins were linearized with 2 mM dithioerythritol (DTE, #D8255, Sigma) at 37 °C for 1 h, and then with 10 mM iodoacetamide (IAM, #I6125, Sigma) at room temperature for 50 min in dark. Next, the linearized proteins were digested to peptides with Lys-C (0.3 µg for 15 µg substrate, #125-05061, Wako) at 37 °C for 3 h and then with Trypsin (0.3 µg for 15 µg substrate, #V5111, Promega) for 16 h. The digested peptides were purified using C18 ZipTip and then labeled by Tandem Mass Tag™ 6-plex (TMTsixplex™, #90064, Thermo Scientific) at room temperature for 1 h. Subsequently, equal amounts of labeled peptides from each group were pooled together and fractionated using high pH reversed-phase chromatography (#84868, Thermo Scientific) with 8 fractions eluted by increased acetonitrile (10, 12.5, 15, 17.5, 20, 22.5, 25, and 50%) buffer in 0.1% triethylamine, before analysis using Orbitrap Elite hybrid mass spectrometer (Thermo Electron, Bremen, Germany).

### Shotgun proteomic identifications and analysis

Mass spectrometric data identifications and analysis were performed as previously described [26]. NanoLC−nanoESI-MS/MS analysis was performed on a nanoAcquity system (Waters, Milford, MA) connected to the Orbitrap Elite hybrid mass spectrometer equipped with a PicoView nanospray interface (New Objective, Woburn, MA). Peptide mixtures were loaded onto a 75 μm ID, 25 cm length C18 BEH column (Waters, Milford, MA) packed with 1.7 μm particles with a pore of 130 Å and were separated using a segmented gradient across 90 min from 5 to 35% solvent B (acetonitrile with 0.1% formic acid) at a flow rate of 300 nl/min and a column temperature of 35 °C. Solvent A was 0.1% formic acid in water. The mass spectrometer was operated in the data-dependent mode. Briefly, survey full scan MS spectra were acquired in the orbitrap (*m/z* 350–1600) with resolution set to 60 K at *m/z* 400 and automatic gain control (AGC) target at 10^6^. The 15 most intense ions were sequentially isolated for HCD MS/MS fragmentation and detection in the orbitrap with previously selected ions dynamically excluded for 60 s. For MS/MS, we used a resolution of 15,000, an isolation window of 2 *m*/*z,* and a target value of 50,000 ions, with maximum accumulation times of 200 ms. Fragmentation was performed with normalized collision energy of 35% and an activation time of 0.1 ms. Ions with single and unrecognized charge state were also excluded.

The identified peptides were then mapped to their corresponding proteins using Proteome Discoverer 2.3 software (Thermo Scientific, MA, USA). Database searching was performed using the Swiss-Prot Mouse database and the Sequest algorithms with settings of 10 ppm precursor mass tolerance as well as 0.02-Da fragment mass tolerance. Carbamidomethylation was defined as a static modification. TMT6plex (N-Terminus), acetylation, and oxidation modifications were defined as dynamic modifications. The results of identified proteins were filtered by medium and high confident peptides with a global false discovery rate <1% based on a target-decoy approach. Only unique peptides for a given protein were considered for the following analysis. For fold change quantification, the ratios of each TMT reporter ion abundance were analyzed using pairwise-ratio-based Student’s *t*-test. Proteins with abundance ratio *p* value < 0.05 were considered as significant.

### Immunoprecipitation

Immunoprecipitation using cell lysates containing equal amounts of proteins was performed as previously described [12]. Briefly, cells were lysed using RIPA lysis buffer containing 50 mM Tris-HCl (pH 7.5), 150 mM NaCl, 1% NP-40, 1% sodium deoxycholate, 0.1% SDS, 1 μg/ml aprotinin, 1 μg/ml leupeptin, 1 mM PMSF, 1 mM sodium vanadate, 4 mM sodium pyrophosphate, and 20 mM NaF. Total cell lysates were incubated with anti-Flag M2 beads (#A2220, Sigma) at 4 °C for 3–4 h, or antibodies conjugated with Protein A-Sepharose (#GE17-0780-01, GE Healthcare) overnight. The beads were washed, and the bound proteins were analyzed by western blotting with various antibodies.

### Ubiquitination assay

In vivo ubiquitination assay was performed by immunoprecipitation as previously described [12]. Briefly, cells were transfected with various constructs, together with HA-ubiquitin, and then treated with 1 μM MG132 for 16 h, before being lysed by RIPA buffer.

### Rac1 activity assay

Detection of active GTP-bound Rac1 was performed as described previously [27]. Cells were lysed using lysis buffer containing 25 mM HEPES (pH 7.5), 150 mM NaCl, 10 mM MgCl2, 1 mM EDTA, 1% Triton X-100, 10% glycerol, 1 μg/ml aprotinin, 1 μg/ml leupeptin, 1 mM PMSF, 1 mM sodium vanadate, 4 mM sodium pyrophosphate, and 20 mM NaF. After lysis and centrifugation, 500 μl of cleared cell lysate was incubated with 10 μg of GST-PAK-CRIB coupled to glutathione–Sepharose 4B beads (#17-0756-05, GE Healthcare) at 4 °C for 45 min. The pulled down complexes were washed with lysis buffer three times and then analyzed using western blot to detect bound Rac1.

### RNA extraction, RT-PCR, and real-time quantitative PCR

RNA was extracted using TRIzol (#15596018, Invitrogen). For cDNA synthesis, 1 µg of total RNA was used with the iScript Reverse Transcription Supermix (#1708840, Bio-Rad). Real-time quantitative PCR (RT-qPCR) was performed using *Power* SYBR Green PCR Master Mix (#4367659, Applied Biosystems) on a LightCycler 480 (Roche). The PCR condition was 95 °C for 15 min, 35 cycles at 94 °C for 15 s, 55 °C for 30 s, and 70 °C for 30 s. Data were normalized to the mean of housekeeping gene *Gapdh*. The primer sequences are *Arhgap17*: Fw 5′-TTTGGTGTGAAGCTAATGGACTT-3′, Rv 5′-TGCTCCAATATGCCCGTAGAA-3′; *Gapdh*: Fw 5′-AGGTCGGTGTGAACGGATTT-3′, Rv 5′-TGTAGACCATGTAGTTGAGGTCA-3′.

### Histology, Nissl staining, immunofluorescence staining, and imaging

Brains were harvested, sectioned, and stained as previously described [28]. Mice were anaesthetized with ice (<P3) or isoflurane (>P3), and then perfused by 4% PFA in phosphate-buffered saline (PBS). The brains were removed, post-fixed in the same fixative at 4 °C overnight, and immersed in 30% sucrose in PBS. Fixed brains were sectioned at a thickness of 60 μm using a vibratome (Leica VT1200S).

For Nissl staining, sections were washed, mounted, and air-dried overnight. The dried sections were re-hydrated in PBS, stained using 0.01% cresyl violet in acetate buffer containing 0.1 M glacial acetic acid and 0.01 M sodium acetate (pH 3.5) at 60 °C for 10 min, rinsed with H_2_O, and then de-stained using 70 and 95% Ethanol for 2 min each. Sections were finally de-hydrated with 100% Ethanol and Xylene before being mounted with Permount (#SP15-500, Fisher Scientific). The stained sections were imaged using an Olympus SZX16 microscope equipped with a 0.5× or 1× objective lens (Olympus) and a DP80 digital camera with a controller software (Olympus). The areas of the cerebellum and EGL were outlined and quantified using ImageJ software (National Institutes of Health, Bethesda, MD, USA). Of note, the EGL area was defined by its strong Nissl staining, which can be distinguished from the weakly stained ML.

For immunofluorescence staining, sections were blocked with 5% goat serum and 0.25% Triton X-100 in PBS for 2 h, incubated with primary antibodies at 4 °C overnight, and then with secondary antibodies and DAPI (#D1306, Invitrogen) at room temperature for 2 h. Incorporated EdU was detected using the Click-iT EdU Kit (#10337, Invitrogen). Sections were washed, mounted with mounting medium (#H-1000, VECTOR), and then stored at 4 °C in dark. The mounted sections were imaged using a confocal laser-scanning microscope (LSM 780, Zeiss) with a 5×, 10×, or 40× objective lens and using ZEN software. Images were quantified and presented using ImageJ software (NIH) or ZEN 2009 Light Edition (Zeiss).

For GNP staining, GNPs on coverslips were permeabilized with cytoskeleton buffer containing 50 mM NaCl, 3 mM MgCl2, 300 mM sucrose, 0.5% Triton X-100, and 10 mM PIPES (pH 6.8) for 20 min. GNPs were then blocked with blocking buffer (10% goat serum and 1% BSA in PBS) for 1 h. GNPs were incubated with primary antibodies in blocking buffer at 4 °C overnight, and then with secondary antibodies in blocking buffer at room temperature for 1 h. Incorporated EdU was detected as described above. Finally, GNPs were washed, mounted with mounting medium, and then stored at 4 °C in dark. Slides were imaged by a Leica SP5 confocal laser-scanning microscope equipped with a 40× objective lens (Leica) and a Leica Application Suite Advanced Fluorescence software.

### Behavioral analyses

All behavioral assays were performed using 2- to 3-month-old male littermates, and genotype-blinded.

### Open field test

The open field apparatus consists of four plexiglass arenas. Each arena is 48 (I) × 48(w) × 35 (h) cm with black-paper-covered floor and walls. Each mouse was placed into a corner of the arena and allowed to explore for 60 min under a standard overhead lighting condition. Their tracks were recorded by a video camera and analyzed using the EthoVision XT software.

### Rotarod test

Mice were placed on a rod of a diameter 3.175 cm (Rota-Rod 47600, Ugo Basile, VA, Italy), which rotated with a constant speed of 4 rpm for 60 s in the training phase, and accelerated from 4 to 40 rpm over 300 s in the testing phase. Their time spent on the rod (latency) were recorded. Mice were given three test trials with 15 min inter-trial intervals. The latency of each mouse was calculated as the mean of three trials and analyzed.

### Grip strength

The forelimb grip strength was measured five times for each mouse using the MK-380CM/R grip strength meter (Muromachi Kikai Co., Tokyo, Japan). The data were averaged and normalized to the body weight of each mouse.

### Spatiotemporal analysis of gait patterns

A commercially available catwalk system was used for acquiring spatiotemporal parameters of mice gait. The catwalk consists of a plexiglass chamber 60 (l) × 6 (w) × 12 (h) cm with a mirror tilted at a 45° angle underneath the walking track so that it reflects the image of the mouse paws. A camera (Panasonic Lumix DC-G9) 40 cm away was used to simultaneously record a side view and an under-view of the mouse paws (mirror image). The catwalk has two LED light arrays achieving an average of 1500 lux.

The mice were placed in the walkway to habituate for at least 30 min before the formal recordings, at which point only one mouse was left inside. The mouse was then allowed to walk naturally from one side to another while being recorded. Walking tasks were recorded for both directions until six satisfactory walks, three per direction, of at least 12 steps without pause were obtained. The videos obtained from each trial were manually analyzed using a Matlab software (MathWorks, version 7.6., R2008a) that came with the gait analysis system [29]. Two videos for each direction were analyzed per mice and averaged, obtaining spatial parameters (step length, stride length, base of support, print length, intermediary toe spread, toe spread, and foot angle), and temporal gait parameters (walking speed, stance/swing phase time, and double support time).

### Grid-walking task

An elevated (40 cm high) 40 × 40 cm wire mesh with grid size 2 × 2 cm was used to assess potential proprioception defects [30, 31]. The experiment was done in the dark (<0.1 Lux on the grid measured with a light meter) and the whiskers from the mice were trimmed the day before experiment, to forbid the compensation of visual and mechanosensing faculties, respectively, on muscle spindle proprioception. An infrared light array (KingNet) was projected at an angle to the grid, allowing the mice to be recorded with the night-shot function of a camera (Sony Handycam FDR-AXP55). The experimental mouse was placed in the center of the mesh and allowed to travel freely for 5 min. Foot faults were then manually counted through video playback software and were defined as a hind paw missing the grid while walking, resulting in the mouse’s ankle sliding fully below the plane of the wire mesh.

### Sample size estimation and statistics

The sample size was determined by using the following formula:

$$n = \frac{{2\sigma ^2\left( {Z_{1 - \beta } + Z_{1 - \frac{\alpha }{2}}} \right)^2}}{{{\Delta}^2}}$$

in which α (probability of type I error) was set to be 0.05, *β* (probability of type II error) was set to be 0.2, *σ* (standard deviation) and △ (difference) were estimated based on our pilot studies. In the histological analyses, *σ* was set to be 8, △ was set to be −20, and thus *n* = 2.51. Therefore, three samples were used in each group. In behavioral assays, *σ* was set to be 23.5, △ was set to be −30, and thus *n* = 9.63. Accordingly, 10 samples were used in each group. No sample was excluded from the analyses and no randomization was used.

Data were analyzed using Prism 9 (Graph Pad) and Excel (Microsoft). Data were first analyzed using Shapiro-Wilk test and Kolmogorov-Smirnov test to know if they normally distributed. Data of two groups following a normal distribution were analyzed using an *F*-test to know the equality of variances between groups. Depending on the results of *F*-test, data of two groups were then analyzed using two-tailed unpaired Student’s *t*-test with or without Welch’s correction. Data of multiple groups were analyzed using one-way ANOVA post hoc Dunnett’s, Turkey, or Holm-Šídák’s test, depending on the recommendation by Prism. Data of two groups not following a normal distribution were analyzed using Mann–Whitney test. *P* value was shown as an exact number, <0.0001, or >0.9999 as Prism presented. *P* < 0.05 was considered as statistically significant.

## Results

### Wdr4 ablation in the central nervous system disrupts cerebellar development and impairs GNP proliferation

**Fig. 1:**
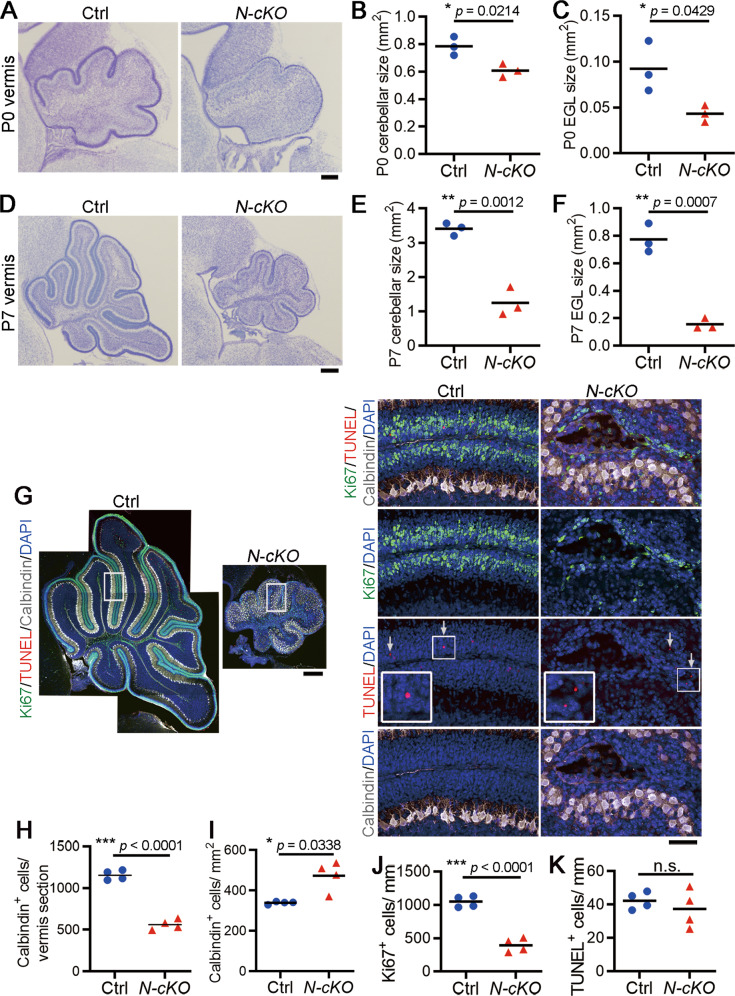
Nervous system-specific knockout of *Wdr4* impairs cerebellar development. **A**–**F** Representative Nissl staining images (**A**, **D**) and quantitative data (**B**, **C**, **E**, **F**) for cerebellar size, foliation, and EGL size in P0 (**A**–**C**) and P7 (**D**–**F**) *Wdr4 N-cKO* and control mice. See also Fig. S1 and Video S1. **G**–**K** Representative confocal images (**G**) and quantitative data (**H**–**K**) showing cerebellum size (**G**), Calbindin^+^ Purkinje neurons (**G**–**I**), Ki67^+^ proliferating GNPs (**G**, **J**), and TUNEL^+^ apoptotic cells (**G**, **K**) in the P7 *Wdr4 N-cKO* and control cerebella. The most left panel in (**G**) was stitched with several images to show the whole cerebellum. The right panels in (**G**) are higher magnification images of the boxed regions in the left panels. Arrows indicate TUNEL^+^ apoptotic cells. The data in (**H**–**I**) were from the whole cerebellar section, and in (**J**–**K**) were from the EGL of lobule V–VI. Scale bars are 150 μm in (**A**), 250 μm in (**D**), (**G**, left), and 50 μm in (**G**, right). Data were from 3 (**B**, **C**, **E**, **F**) or 4 (**H**–**K**) cerebella in each group and analyzed using two-tailed unpaired Student’s *t*-test without Welch’s correction (equal variances, **B**, **C**, **E**, **F**, **H**, **J**, **K**) or with Welch’s correction (unequal variances, **I**), *p* = 0.0214 in (**B**), 0.0429 in (**C**), 0.0012 in (**E**), 0.0007 in (**F**), <0.0001 in (**H**), 0.0338 in (**I**), <0.0001 in (**J**), and 0.4951 in (**K**). Data are represented as individual points and mean; *p < 0.05, **p < 0.005, ***p < 0.0005; n.s., non-significant.

The embryos of *Wdr4*^−/−^ mice exhibit abnormal brain morphology and result in embryonic death around E10.5 [18]. For this reason, and in order to explore the functions of Wdr4 in brain development, *Wdr4*^*flox/flox*^ mice [12, 18] were crossed with *Nestin-Cre; Wdr4*^*flox/+*^ mice to delete *Wdr4* gene predominantly in the central nervous system [19]. While no difference was observed by comparing *Nestin-Cre; Wdr4*^*flox/+*^ (termed “control” hereafter) with WT mice, *Nestin-Cre; Wdr4*^*flox/flox*^ (termed “*Wdr4 N-cKO*” hereafter) mice showed an impaired gait phenotype starting around P14 (Video S1) and died around P21 (pre-weaning lethality; 41/41 pups from 23 litters), and western blot analysis confirmed the lack of Wdr4 expression in the *Wdr4 N-cKO* cerebella (Fig. S1A). Given the obvious locomotion defects, we focused on the function of Wdr4 in cerebellum development. To investigate the histological changes in cerebellum, P0 and P7 brains were harvested, sectioned, and subjected to Nissl staining. Histological images and quantifications of the P0 sagittal sections showed a ~23% decrease in size, a significantly reduced foliation of the cerebellar vermis (Fig. 1A, B), and a ~53% reduction of the EGL area (Fig. 1A, C) in *Wdr4 N-cKO* compared to those of the control. Yet, the abnormal phenotypes were more severe at P7, where the sizes of cerebellum and EGL decreased by ~63% and ~80%, respectively, in the *Wdr4 N-cKO* vermis compared to those in the controls (Fig. 1D–F). Similarly, there were decreases of ~40% and ~78%, respectively, in the *Wdr4 N-cKO* hemisphere size (Fig. S1B–D). In addition, the Calbindin^+^ Purkinje neurons showed a disorganized alignment (Fig. 1G), a ~51% decrease in number (Fig. 1G, H), and a ~40% increase in density (Fig. 1G, I), in the P7 *Wdr4 N-cKO* cerebella compared to the controls. These results indicate that Wdr4 positively regulates the cerebellum and EGL development.

To understand whether the reduced EGL size is due to a deficiency in GNP proliferation or an increase in apoptosis, both immunofluorescence staining for a cell cycle marker Ki67 and terminal deoxynucleotidyl transferase dUTP nick end labeling (TUNEL) were performed. Confocal images and quantifications showed a ~62% decrease of the Ki67 index (number of Ki67^+^ cells/mm) in the P7 *Wdr4 N-cKO* cerebella compared to the controls (Fig. 1G, J), while no difference in the number of TUNEL^+^ apoptotic cells was observed (Fig. 1G, K). These findings indicate that Wdr4 promotes EGL development by increasing GNP proliferation rather than suppressing their apoptosis.

### Wdr4 promotes cerebellar GNP proliferation in a cell-autonomous manner

**Fig. 2:**
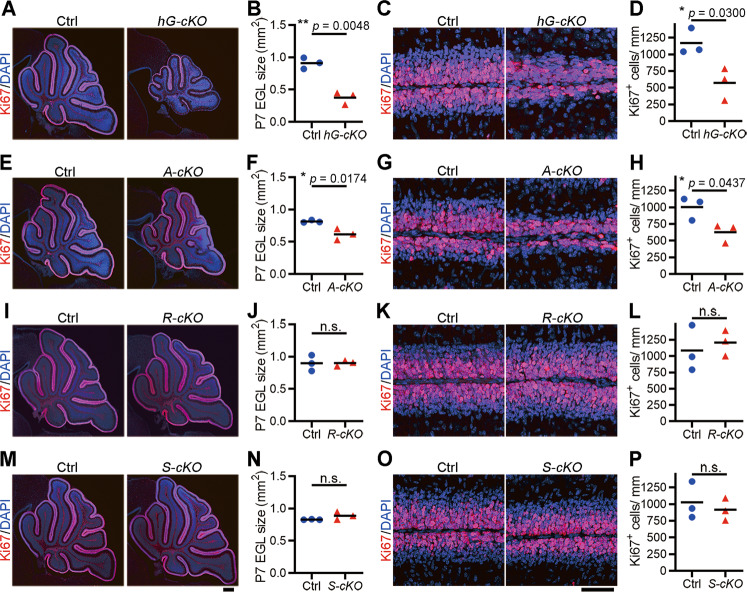
*Wdr4* ablation impairs GNP proliferation in a cell-autonomous manner. **A**–**P** Representative confocal images and quantitative data from the whole cerebellar section (**A**, **B**, **E**, **F**, **I**, **J**, **M**, **N**), and the EGL of lobule V–VI (**C**, **D**, **G**, **H**, **K**, **L**, **O**, **P**) showing EGL size and Ki67^+^ proliferating GNPs in the indicated *Wdr4* conditional knockout and control mice at P7 (scale bars, 250 μm in the left and 50 μm in the right). Data were from 3 cerebella in each group and analyzed using a two-tailed unpaired Student’s *t*-test without Welch’s correction (equal variances, **B**, **D**, **F**, **H**, **J**, **L**, **P**) or with Welch’s correction (unequal variances, **N**), *p* = 0.0048 in (**B**), 0.0300 in (**D**), 0.0174 in (**F**), 0.0437 in (**H**), 0.9848 in (**J**), 0.6309 in (**L**), 0.2248 in (**N**), and 0.5906 in (**P**). Data are represented as individual points and mean; *p < 0.05, **p < 0.005; n.s., non-significant. See also Figs. S2–4.

During cerebellum development, the proliferation of GNPs is controlled by both intrinsic programs and extrinsic factors derived from their neighboring cells [2, 3]. By retrieving data from a previous single-cell RNA-seq study [32], we found that *Wdr4* is expressed in all major cell types in the cerebellum, including granule neurons, Purkinje neurons, interneurons, and Bergmann glia (Fig. S2). To examine the origin of the cell type whose Wdr4 deficiency leads to the reduced cerebellar EGL, *Wdr4*^*flox/flox*^ mice were individually crossed with *Cre* mouse lines driven by different cell type-specific promoters. First, *hGFAP-Cre* was used to delete *Wdr4* in radial glia (i.e., the transitional progenitors between neural stem cells and mature neurons) and Bergmann glia, but not in Purkinje neurons [20]. The cerebella of the resulting *hGFAP-Cre; Wdr4*^*flox/flox*^ (termed “*Wdr4 hG-cKO”* hereafter) and *hGFAP-Cre; Wdr4*^*flox/+*^ (termed “control”) mice were analyzed at P7. A ~55% reduction of the EGL size and a ~51% reduction of the Ki67 index were found in *Wdr4 hG-cKO* compared to those of the control group (Fig. 2A–D). Western blot confirmed no expression of Wdr4 in the GNPs isolated from the *Wdr4 hG-cKO* cerebella (Fig. S3A). Next, in order to ablate the *Wdr4* gene in cerebellar GNPs, *Atoh1-Cre* [21] was used. The EGL size and Ki67 index both decreased by ~25% and ~38%, respectively, in the P7 *Atoh1-Cre; Wdr4*^*flox/flox*^ (termed *Wdr4 A-cKO* hereafter) cerebella compared to those in the control group (*Atoh1-Cre; Wdr4*^*flox/+*^) (Fig. 2E–H). On the other hand, *Wdr4* deletion in Purkinje neurons using *Rora-Cre (Wdr4 R-cKO)* [22], or in Bergmann glia using *Sept4-Cre* (*Wdr4 S-cKO*, Founder line *OX54-CRE*, the Gene Expression Nervous System Atlas Project), resulted in no difference in EGL size and Ki67 index compared to their controls (Fig. 2I–P). Immunostaining showed the lack of expression of Wdr4 in Purkinje neurons or Bergmann glia in the cerebellum of *Wdr4 R-cKO* or *Wdr4 S-cKO*, respectively (Figs. S3B and S3C), thus excluding the possibility of Wdr4 having a role in controlling the expansion of the GNP population through either the Purkinje neurons or Bergmann glia. The weaker phenotype seen in the *Wdr4 A-cKO* compared to *Wdr4 hG-cKO* mice is likely due to the expression of Atoh1-Cre at a later cell lineage compared to hGFAP-Cre [20, 21]. In addition, the phenotype of reduced EGL in the cerebellum of P7 *Wdr4 A-cKO* was only observed in lobules I-VI, but not in lobules VII-X (Figs. S4A and S4B), consistent with an enriched expression of Wdr4 in the lobule I-VI (Fig. S4C). Together, these data support a cell-autonomous role of Wdr4 in promoting cerebellar GNP proliferation.

### Wdr4 increases the number of proliferating GNPs by inhibiting their exit from the cell cycle

**Fig. 3:**
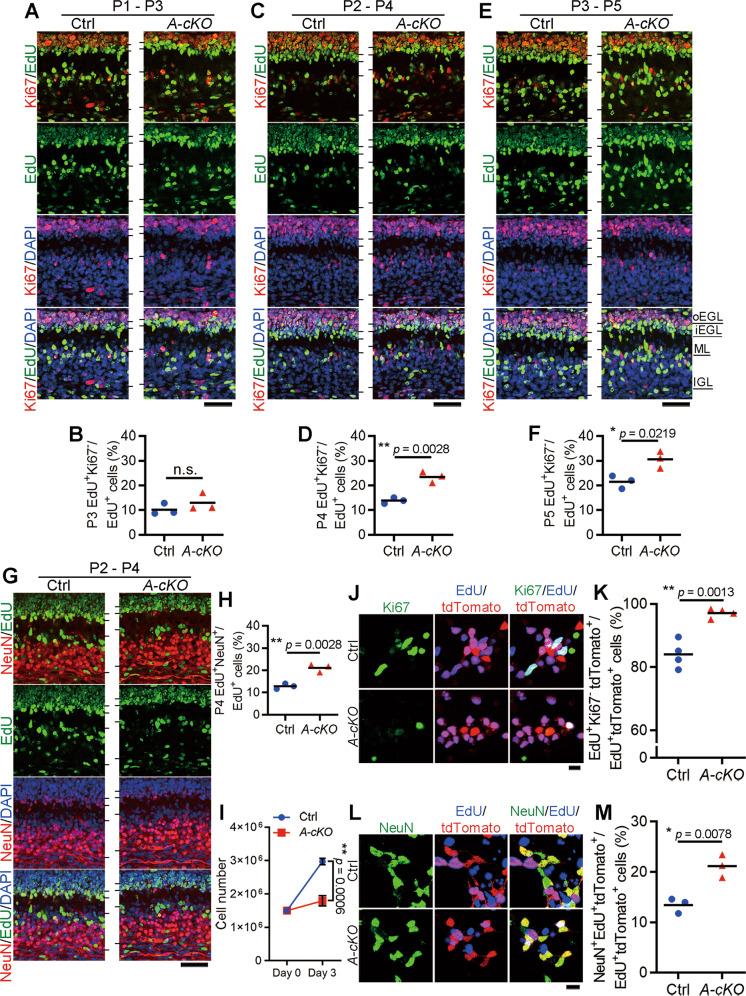
*Wdr4* deficiency in cerebellar GNPs accelerates cell cycle exit. **A**–**F** Representative confocal images (**A**, **C**, **E**) and quantitative data (**B**, **D**, **F**) from lobule V for examining GNP cell cycle exit in the *Wdr4 A-cKO* and control cerebella labeled by EdU at P1, P2, or P3 and harvested 2 days later for staining. The Ki67^+^ proliferating GNPs mainly located in the outer EGL (oEGL), while the cell cycle exit population (EdU^+^Ki67^-^) mostly located in the inner EGL (iEGL), ML, and IGL. **G**–**H** Representative confocal images (**G**) and quantitative data (**H**) from lobule V for measuring GNP differentiation into NeuN^+^ granule neurons in the *Wdr4 A-cKO* and control cerebella labeled by EdU at P2 and harvested 2 days later for staining. **I** Quantitative data measuring proliferation in GNPs isolated from P7 *Wdr4 A-cKO* and control mice, cultured for 3 days. See also Fig. S5. **J**–**K** Representative confocal images (**J**) and quantitative data (**K**) for measuring cell cycle exit in P7 *Wdr4 A-cKO; Ai14* and control GNP cultures stained at DIV3. **L**–**M** Representative confocal images (**L**) and quantitative data (**M**) for measuring differentiation into NeuN^+^ granule neurons in P7 *Wdr4 A-cKO; Ai14* GNP cultures and controls stained at DIV3. Scale bars are 50 μm in (**A**, **C**, **E**, **G**) and 25 μm in (**J**, **L**). Data were from 3 (**A**–**H**, **L**–**M**) or 4 (**I**–**K**) cerebella in each group and analyzed using two-tailed unpaired Student’s *t*-test without Welch’s correction (equal variances), *p* = 0.3160 in (**B**), 0.0028 in (**D**), 0.0219 in (**F**), 0.0028 in (**H**), 0.0006 in (**I**), 0.0013 in (**K**), and 0.0078 in (**M**). Data are represented as individual points and mean, or mean ± S.E.M.; *p < 0.05, **p < 0.005; n.s., non-significant.

The number of proliferating cerebellar GNPs is determined by their cell cycle entry and exit. Their entry starts around E17.5, right after their arrival onto the EGL. After the first round of the cell cycle, the divided GNPs can continue to undergo another round to produce more GNPs, or exit the cell cycle to start migration into the IGL, where they differentiate and become NeuN^+^ granule neurons [3]. We reasoned that the reduced number of proliferating GNPs seen in the cerebella of *Wdr4 A-cKO* mice may be resulted from an increased tendency of GNPs to exit the cell cycle. To test this hypothesis, *Wdr4 A-cKO* and control mice at P1, P2, or P3 were subcutaneously injected over the cerebellum with 5-ethynyl-2’deoxyuridine (EdU). The cerebella were then harvested 48 h after injection, since the estimated length of GNP cell cycle is ~2 days during P3-6 [33]. Thus, the EdU^+^Ki67^−^ cells represent GNPs’ progeny that have exited the cell cycle. Although GNPs labeled at P1 in both *Wdr4 A-cKO* and control mice showed no difference in cell cycle exits (Fig. 3A, B), *Wdr4 A-cKO* GNPs labeled at P2 or P3 exhibited ~69% and ~42% increases, respectively, in EdU^+^Ki67^-^/EdU^+^ population compared to those in the control mice (Fig. 3C–F). An accompanied ~64% increase of EdU^+^NeuN^+^/EdU^+^ granule neuron numbers was detected in the P4 *Wdr4 A-cKO* cerebella (labeled by EdU at P2) compared to the control group (Fig. 3G, H). These findings suggest that *Wdr4* deficiency in GNPs promotes cell cycle exit, instigating the precocious differentiation of granule neurons.

To substantiate the role of Wdr4 in controlling GNP cell cycle exit, we tested whether this effect could be recapitulated in an in vitro system. To this end, GNPs were isolated from P7 cerebella of *Wdr4 A-cKO* and control mice using Percoll density gradient [25], and ~85% of the isolated cells expressed the GNP marker Atoh1 (Fig. S5). The isolated GNPs were labeled immediately by EdU, cultured and harvested after 3 days in vitro (DIV) to count their cell numbers for proliferation analysis. A ~40% decrease of total cell number at DIV3 was found in the GNPs from *Wdr4 A-cKO* cerebella compared to the controls (Fig. 3I). For a more precise calculation, the fluorescence reporter mice *Ai14*^*flox/flox*^ [23], in which tdTomato is expressed in Cre^+^ lineage cells, were crossed with *Wdr4*^*flox/flox*^ mice. Next, *Atoh1-Cre; Wdr4*^*flox/+*^ mice were crossed with *Wdr4*^*flox/flox*^; *Ai14*^*flox/flox*^ mice to obtain *Atoh1-Cre; Wdr4*^*flox/+*^; *Ai14*^*flox/+*^ (control) and *Atoh1-Cre; Wdr4*^*flox/flox*^; *Ai14*^*flox/+*^ (*Wdr4 A-cKO; Ai14*) mice. Consistent with the previous study [34], the tdTomato signal indicated that Atoh1-Cre was expressed in the EGL of every lobule at P0 (Fig. S6A). Accordingly, immunostaining showed the devoid of Wdr4 expression in the EGL of P0 *Wdr4 A-cKO* cerebella (Fig. S6B). GNPs were isolated from these mice to monitor their cell cycle exit using EdU labeling followed by Ki67 staining; only the tdTomato^+^ cells were counted for. We found a ~15% increase of the EdU^+^Ki67^-^/EdU^+^ (Figs. 3J, K), and a ~57% increase of the EdU^+^NeuN^+^/EdU^+^ (Fig. 3L, M) populations in the GNPs from *Wdr4 A-cKO; Ai14* cerebella compared to their controls. Thus, in vitro data demonstrated that *Wdr4* deficiency in GNPs presented a decrease in proliferation, while showing increased cell cycle exit and differentiation. Together, these results reinforce Wdr4’s role in inhibiting GNP cell cycle exit and promoting their expansion in a cell–autonomous manner.

### Wdr4 deficiency reduces IGL and ML sizes and impairs Purkinje neuron organization

**Fig. 4:**
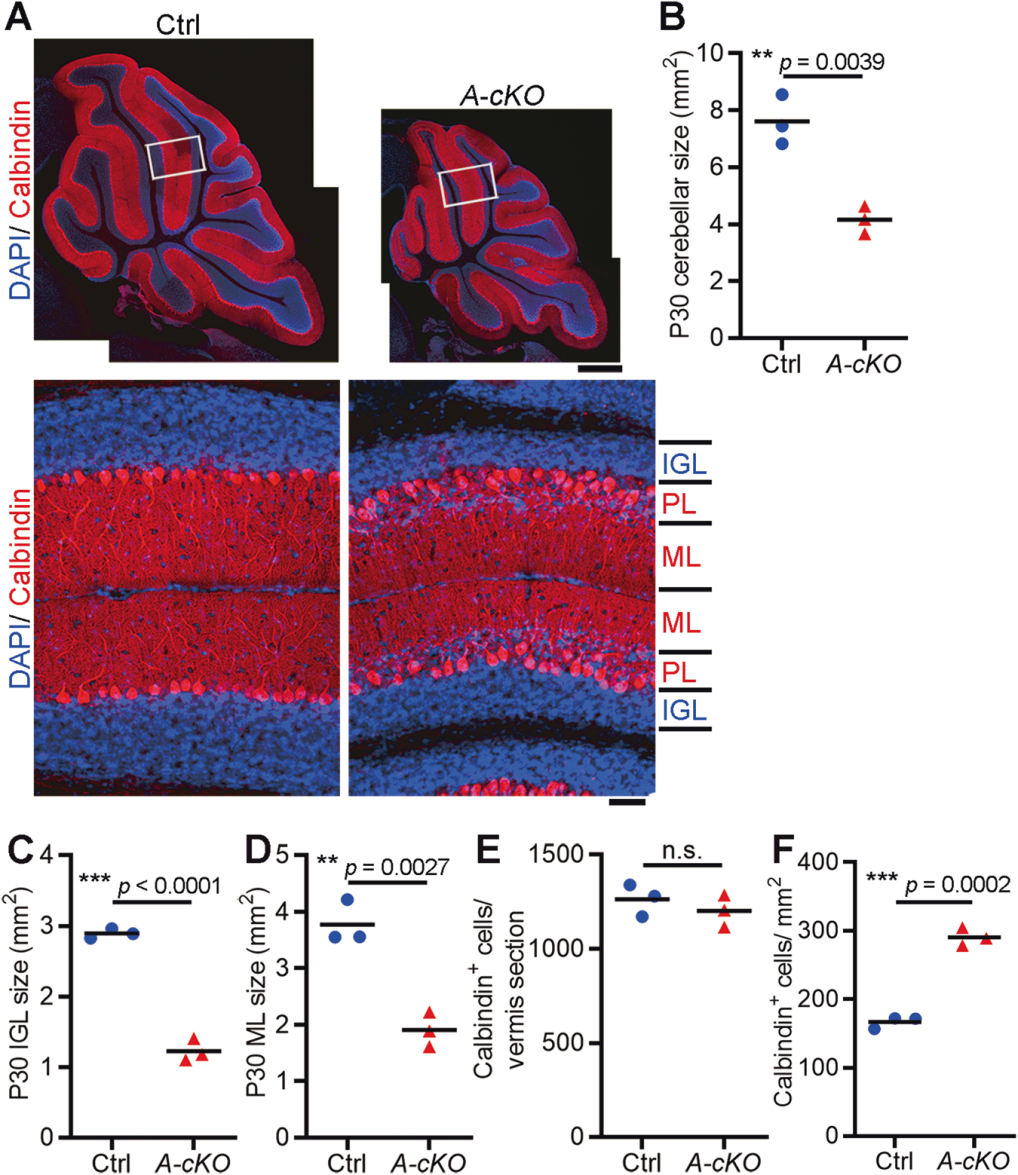
*Wdr4* deficiency in cerebellar GNPs leads to IGL and ML size reduction and Purkinje neuron disorganization. **A**–**F** Representative confocal images (**A**) and quantitative data (**B**–**F**) showing the sizes of cerebellum (**A**, **B**), IGL (**A**, **C**), ML (**A**, **D**), and the number (**A**, **E**) as well as the density (**A**, **F**) of Purkinje neurons, in the P30 *Wdr4 A-cKO* and control mice. The upper-left panel in (**A**) was stitched with several images to display the whole cerebellum. The lower panels in (**A**) are higher magnification images of the boxed regions in the upper panels, showing disorganized Purkinje neurons in P30 *Wdr4 A-cKO* cerebella (scale bars, 500 μm in the upper panels and 50 μm in the lower panels). Data were from 3 cerebella in each group and analyzed using two-tailed unpaired Student’s *t*-test without Welch’s correction (equal variances), *p* = 0.0039 in (**B**), < 0.0001 in (**C**), 0.0027 in (**D**), 0.4276 in (**E**), and 0.0002 in (**F**). Data are represented as individual points and mean; **p < 0.005, ***p < 0.0005; n.s., non-significant.

Postmitotic granule neurons exit the EGL and migrate inward to form the IGL, where they become fully mature, in a process that continues until ~P15 [2, 4]. To determine whether the decreased GNP proliferation upon *Wdr4* deletion results in the reduction of mature granule neurons and/or IGL when cerebellum development is completed, P30 *Wdr4 A-cKO* and control cerebella were harvested, sectioned, and stained with 4’,6-diamidino-2-phenylindole (DAPI) to label different layers in the cerebellum. A reduction of ~45% in cerebellar size (Figs. 4A and 4B), including size reductions of ~58% for the IGL (Figs. 4A and 4C) and ~49% for the ML (Figs. 4A and 4D), was found in P30 *Wdr4 A-cKO* compared to those of the controls. In addition, while there was no difference in the number of Purkinje neurons (Figs. 4A and 4E), we found a ~74% increase in their density (Figs. 4A and 4F) and a concomitant disorganization of their alignment (Fig. 4A, lower panels) in the P30 *Wdr4 A-cKO* compared to the controls. These results demonstrate the crucial role of Wdr4 in maintaining the sizes of the IGL, ML and the organization of Purkinje neurons, where the latter two phenotypes are mediated by a non-cell-autonomous mechanism.

### Wdr4 promotes coordinated locomotion

**Fig. 5:**
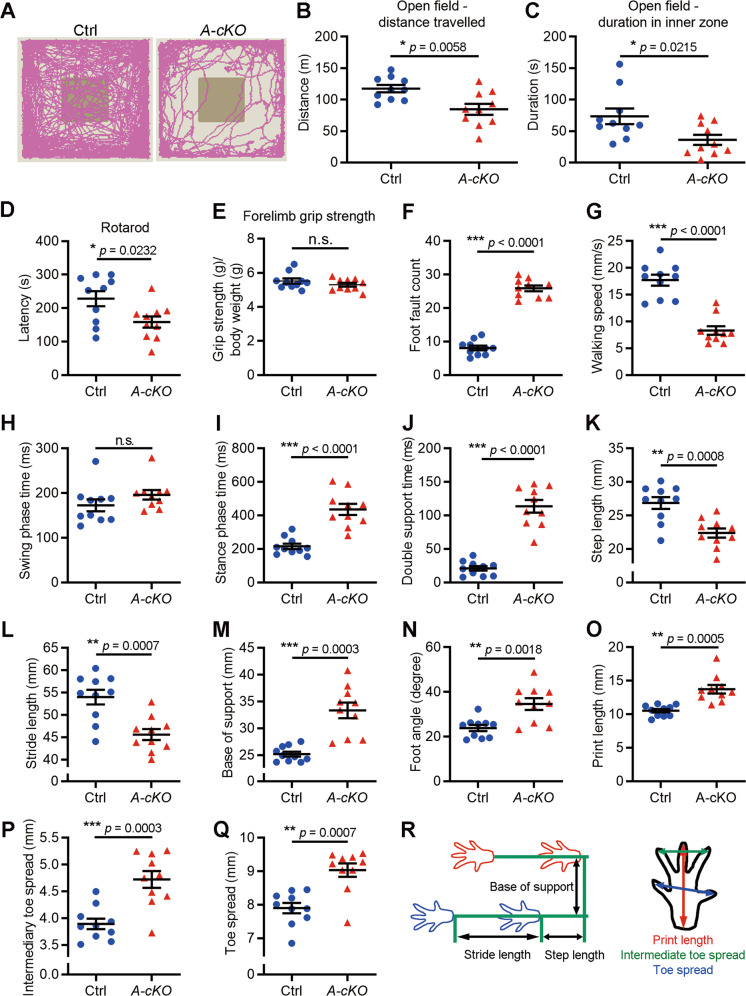
*Wdr4* ablation in cerebellar GNPs impairs locomotion and coordination. **A**–**C** Representative moving traces (**A**) and quantitative data (**B**, **C**) for the moving distance (**B**) and time spent in the inner zone (**C**) in an open field test for *Wdr4 A-cKO* and control mice. **D** Quantitative data for latency to fall in rotarod test for *Wdr4 A-cKO* and control mice. **E** Quantitative data for grip strength in *Wdr4 A-cKO* and control mice. **F** Quantitative data for foot faults in grid-walking test for *Wdr4 A-cKO* and control mice. **G**–**Q** Quantitative data for walking speed (**G**), step length (**K**), stride length (**L**), stance phase time (**I**), double support time (**J**), base of support (**M**), foot angle (**N**), print length (**O**), intermediary toe spread (**P**), and toe spread (**Q**) in gait analyses for *Wdr4 A-cKO* and control mice. **R** Schematic representation of the parameters measured in gait analysis. Data were from 10 adult male mice (2–3 months old) in each group. Data following a normal distribution were analyzed using two-tailed unpaired Student’s *t*-test with Welch’s correction (unequal variances, **I**, **J**, **M**, **O**) or without Welch’s correction (equal variances, **B**–**G**, **K**, **L**, **N**, **P**), depending on the F-test results. Data not following a normal distribution were analyzed using Mann–Whitney test (**H**, **Q**). *p* = 0.0058 in (**B**), 0.0215 in (**C**), 0.0232 in (**D**), 0.2493 in (**E**), <0.0001 in (**F**), < 0.0001 in (**G**), 0.0605 in (**H**), < 0.0001 in (**I**), < 0.0001 in (**J**), 0.0008 in (**K**), 0.0007 in (**L**), 0.0003 in (**M**), 0.0018 in (**N**), 0.0005 in (**O**), 0.0003 in (**P**), and 0.0007 in (**Q**). Data are represented as individual points and mean ± S.E.M.; *p < 0.05, **p < 0.005, ***p < 0.0005; n.s., non-significant. See also Videos S2A and S2B.

The cerebellum is responsible for locomotor functions [1]. To test if the reduced size of the cerebellum in *Wdr4 A-cKO* mice would result in locomotive abnormalities, a series of behavioral assays were performed on adult mice (2–3 months old). First, in the open field test, *Wdr4 A-cKO* mice showed a ~28% reduction of the total traveling distance and a ~51% reduction of time spent in the inner zone, compared to control mice (Fig. 5A–C). Likewise, a ~30% decrease in latency was observed in the rotarod test (Fig. 5D), revealing a similar impairment of the *Wdr4 A-cKO* mice in motor activity. Importantly, the two groups performed similarly in the grip test (Fig. 5E), thus excluding a defect in the muscle strength. To analyze motor coordination, we performed a grid-walking test [31], where *Wdr4 A-cKO* mice showed a ~2.2 times increase in their number of foot faults compared to the control mice (Fig. 5F). Furthermore, we observed abnormal gait phenotypes (duck feet and upright tails; Videos S2A and S2B) in *Wdr4 A-cKO* mice. Further analyses of gait temporal parameters revealed a ~53% decrease in the walking speed of *Wdr4 A-cKO* mice (Fig. 5G). While no difference was found in swing phase time (Fig. 5H), stance phase time and double support time (when both feet are in contact with the ground simultaneously) were increased ~1 time (Fig. 5I) and ~4.4 times (Fig. 5J), respectively, in *Wdr4 A-cKO* mice compared to the controls. In the spatial parameters, consistent with the decreased walking speed, reductions of ~17% in step length (Fig. 5K, R) and ~16% in stride length (Fig. 5L, R) were found in *Wdr4 A-cKO* mice compared to the control group. Furthermore, increasements of ~32% in base of support (horizontal stride width during the double-support phase; Fig. 5M, R), ~45% in foot angle (angle of external rotation of the foot; Fig. 5N), ~30% in print length (distance from the third toe to the heel; Fig. 5O, R), ~21% in intermediary toe spread (distance from the second to the fourth toe; Fig. 5P, R), and ~14% in toe spread (distance from the first to the fifth toe; Fig. 5Q, R) were detected in *Wdr4 A-cKO* mice compared to the controls. Thus, Wdr4 is essential for maintaining normal gait cycles in both the spatial and temporal dimensions. Together, these behavior analyses indicate that Wdr4 is crucial for both locomotion and motor coordination.

### Wdr4 promotes Arhgap17 ubiquitination and degradation to activate Rac1

**Fig. 6:**
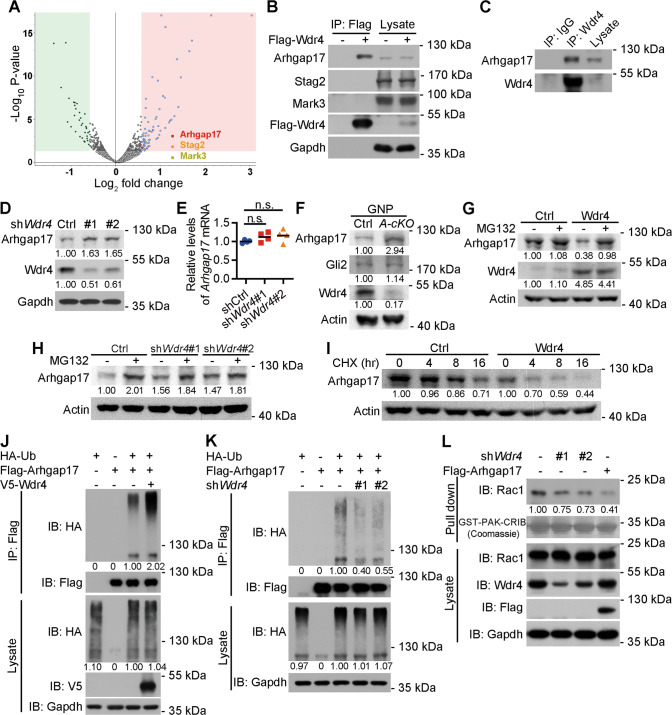
Wdr4 promotes the ubiquitination and degradation of Arhgap17 to activate Rac1. **A** The Volcano plot of proteins identified by LC-MS/MS from GNPs isolated from the P7 *Wdr4 A-cKO; Ai14* and control cerebella. Differentially expressed proteins (*p* < 0.05 and fold change > 1.5) are marked in blue (for up-regulated) and green (for down-regulated). Among the upregulated proteins, those with a known anti-proliferative function are marked in red, orange or yellow with their names. Data were from 3 cerebella in each group and were analyzed using pairwise-ratio-based, Student’s *t*-test by the Proteome Discoverer 2.3 software. **B**, **C** Immunoprecipitation analysis for the interaction between exogenous (**B**) or endogenous (**C**) Wdr4 with indicated proteins. **D**–**E** Western blot (**D**) and RT-qPCR (**E**) analyses for the expression of Arhgap17 protein or *Arhgap17* mRNA in N2a cells expressing control or *Wdr4* shRNAs. Data in (**E**) were from four repeats in each group and analyzed using one-way ANOVA post hoc Dunnett’s test, *p* = 0.3302 in sh*Wdr4*#1 v.s. shCtrl, and 0.1855 in sh*Wdr4*#2 v.s. shCtrl. Data are represented as individual points and mean. **F** Western blot analysis for Arhgap17 and Gli2 expression in the purified GNPs from the P7 *Wdr4 A-cKO* and control cerebella. **G**, **H** Western blot analysis for Arhgap17 expression in N2a cells stably expressing Wdr4 (**G**) or Wdr4 shRNAs (**H**) and treated with 1 μM MG132 for 16 h. **I** Western blot analysis for Arhgap17 expression in N2a cells stably expressing Wdr4 and treated with 100 μg/ml CHX for indicated time points. The levels of Arhgap17 were normalized to the 0 h time point in the control or Wdr4-expressing group, respectively, and indicated at the bottom. **J**, **K** In vivo ubiquitination assay using N2a cells (**J**) or N2a cells expressing Wdr4 shRNAs (**K**) and transfected with indicated constructs. **L** Rac1 activity assay using N2a cells expressing Wdr4 shRNAs or transfected with Flag-Arhgap17. The western blot results were quantified using ImageJ software. The protein levels were normalized first to the Gapdh protein level in each group and then to the corresponding control groups, and expressed as fold changes at the bottom. All western blot analyses were done at least twice.

To understand the molecular mechanism by which Wdr4 promotes GNP proliferation and thus contributes to cerebellar development, we thought to investigate the proteome alterations mediated by Wdr4 deletion in GNPs. To do this, P7 cerebellar GNPs were isolated using fluorescence-activated cell sorting (FACS) of tdTomato^+^ cells from the cerebellar lobule I-VI of 3 *wdr4 A-cKO; Ai14* and 3 control mice, since the effect of Wdr4 on GNP proliferation was obvious in these lobules (Figs. S4A and S4B). Proteins were extracted from the isolated GNPs, digested by Trypsin and Lys-C, labeled by different tandem mass tags (TMT), and fractionated before running them through liquid chromatography-tandem mass spectrometry (LC-MS/MS). A total of 3504 proteins were identified in the quantitative proteomics analysis. Among them, 74 proteins were upregulated and 34 proteins were down-regulated (*p* < 0.05 and fold change >1.5 or <0.666) (Fig. 6A and Table S1). Among the upregulated proteins, Arhgap17 [35], Stag2 [36], and Mark3 [37] were reported to inhibit cell proliferation (Fig. 6A, red, orange, and lemon dot, respectively). To test if they interact with Wdr4, an in vivo binding assay was performed using N2a mouse neuroblastoma cells. Wdr4 fused with a Flag tag was ectopically expressed and immunoprecipitated using agarose beads conjugated with anti-Flag antibodies. While Stag2 and Mark3 were not detected in the immunoprecipitants, Arhgap17 was co-immunoprecipitated with Flag-Wdr4 (Fig. 6B). In addition, endogenous Arhgap17 was detected in the complex immunoprecipitated by an antibody against endogenous Wdr4 (Fig. 6C), indicating that Wdr4 and Arhgap17 interact endogenously. Based on these findings, we focused the following studies on Arhgap17.

Next, we determined whether manipulation of Wdr4 expression could regulate Arhgap17 expression. Knockdown of *Wdr4* in N2a cells increased Arhgap17 protein, but not mRNA, levels (Fig. 6D, E). Similarly, *Wdr4 A-cKO* GNPs exhibited a higher Arhgap17 protein expression level compared to their controls (Fig. 6F). However, the expression of Gli2, one of the downstream effectors of the Shh pathway [38], was comparable in the *Wdr4 A-cKO* and control GNPs (Fig. 6F). Of note, western blot results showed a low, but detectable level of Wdr4 protein expression in the *Wdr4 A-cKO* GNPs (Fig. 6F), which is likely due to the ~85%, rather than 100%, purity of the GNP isolation (Fig. S5). Conversely, Arhgap17 protein expression levels were decreased by the overexpression of Wdr4 in N2a cells (Fig. 6G). To test whether the change of Arhgap17 protein levels was mediated by proteasomal degradation (a destination of K48-linked poly-ubiquitinated proteins), N2a cells were treated with proteasome inhibitor MG132 before being harvested and analyzed by western blot. The Wdr4-mediated Arhgap17 protein changes were abolished upon treatment with MG132 (Fig. 6G, H), suggesting that these changes are mediated through proteasomal degradation. In addition, treatment with cycloheximide, a protein synthesis inhibitor, revealed that the degradation of Arhgap17 was increased with the overexpression of Wdr4 (Fig. 6I). Finally, Arhgap17 ubiquitination was increased by Wdr4 overexpression (Fig. 6J), and decreased by *Wdr4* knockdown (Fig. 6K), as indicated by in vivo ubiquitination assays on N2a cells. Together, these results support the idea that Wdr4 promotes the ubiquitination and proteasomal degradation of Arhgap17.

Arhgap17 is a GTPase-activating protein (GAP) that acts on Rac1 to facilitate its inactivation [39]. The decreased Rac1 activity is known to promote cell cycle exit [40–42]. Thus, to test if Rac1 activity is regulated by Wdr4, GST-PAK-CRIB was used to pull down GTP-bound Rac1 (the active form) from lysates and subsequently analyzed by western blot. We found that Wdr4 knockdown in N2a cells decreased Rac1 activity (Fig. 6L). These results suggest that Wdr4-mediated Arhgap17 degradation promotes Rac1 activation to support GNP proliferation.

### Arhgap17 downregulation and Rac1 activation contribute to the inhibitory effect of Wdr4 on cerebellar GNP cell cycle exit

**Fig. 7:**
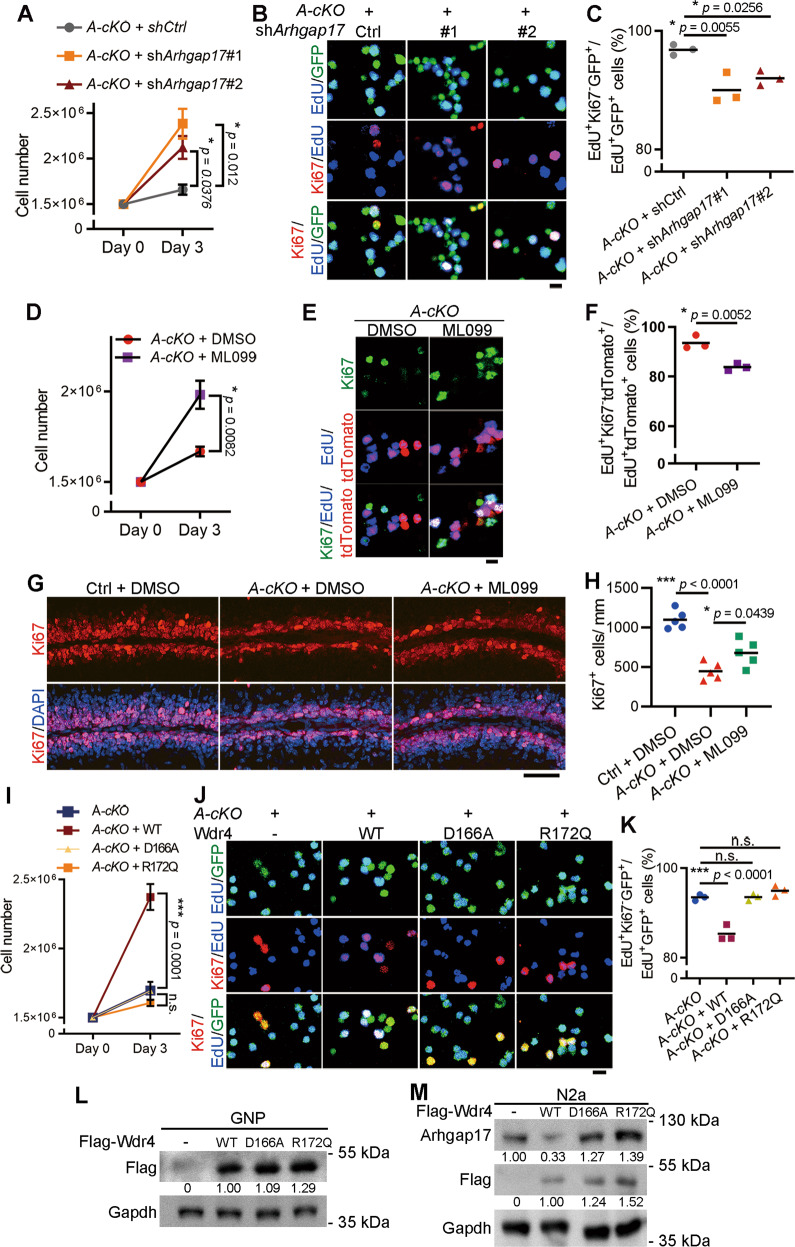
*Arhgap17* knockdown or Rac1 activation rescues the GNP proliferation defect caused by *Wdr4* deletion. **A**, **D**, **I** Quantitative data measuring proliferation in GNPs isolated from P7 *Wdr4 A-cKO* mice and infected with lentiviruses carrying GFP and shRNA against *Arhgap17* or control (**A**), treated with Rac1 activator ML099 or DMSO (**D**), or infected with lentiviruses carrying GFP and Wdr4 WT or mutants (**I**) at DIV 0. The capability of *Arhgap17* shRNAs to knockdown the expression of *Arhgap17* is shown in Fig. S7. The total cell numbers at DIV 0 and DIV 3 were counted. Data were from 3 (**A**, **I**) or 6 (**D**) cerebella in each group and analyzed using one-way ANOVA post hoc Holm-Šídák’s test (**A**), two-tailed unpaired Student’s *t*-test with Welch’s correction (unequal variances, **D**), or one-way ANOVA post hoc Dunnett’s test (**I**). *p* = 0.012 in sh*Arhgap17*#1 v.s. shCtrl, and 0.0376 in sh*Arhgap17*#2 v.s. shCtrl (**A**), *p* = 0.0082 in (**D**), *p* = 0.0001 in Wdr4 WT, >0.9999 in Wdr4 D166A, and 0.5937 in Wdr4 R172Q, compared to the control group (**I**). **B**, **C**, **J**, **K** Representative confocal images (**B**, **J**) and quantitative data (**C**, **K**) for measuring cell cycle exit in P7 *Wdr4 A-cKO* GNP cultures with *Arhgap17* knockdown (**B**, **C**) or overexpression of WT or mutant Wdr4 (**J**, **K**). The GNPs were isolated, treated with EdU for 2 h, infected with lentiviruses carrying GFP and shRNA or cDNA at DIV 0, and harvested for staining analysis at DIV 3. Data were from 3 cerebella in each group and analyzed using one-way ANOVA post hoc Dunnett’s test. *p* = 0.0055 in sh*Arhgap17*#1 v.s. shCtrl, and 0.0256 in sh*Arhgap17*#2 v.s. shCtrl (**C**), *p* < 0.0001 in Wdr4 WT, >0.9999 in Wdr4 D166A, and 0.3568 in Wdr4 R172, compared to the control group (**K**). **E**, **F** Representative confocal images (**E**) and quantitative data (**F**) for measuring cell cycle exit in P7 *Wdr4 A-cKO; Ai14* GNP cultures treated with ML099 or DMSO at DIV 0 and harvested 3 days later. The GNP cultures were treated with EdU for 2 h before ML099 treatment. Data were from 3 cerebella in each group and analyzed using two-tailed unpaired Student’s *t*-test without Welch’s correction (equal variances), *p* = 0.0052. **G**, **H** Representative confocal images (**G**) and quantitative data (**H**) from the EGL of lobule V–VI measuring the number of Ki67^+^ proliferating GNPs in the P7 *Wdr4 A-cKO* or control cerebella. The *Wdr4 A-cKO* and control mice were injected once with DMSO or ML099 (20 mg/kg) at P3, and harvested 4 days later. Data were from 5 cerebella in each group and analyzed using one-way ANOVA post hoc Turkey’s test, *p* < 0.0001 in (**H**, *A-cKO* v.s. Ctrl with DMSO), and =0.0439 in (**H**, ML099 v.s. DMSO in *A-cKO*). See also Fig. S8. Scale bars are 25 μm in (**B**, **E**, **J**) and 50 μm in (G). Data are represented as individual points and mean, or mean ± S.E.M.; *p < 0.05, ***p < 0.0005; n.s., non-significant. **L** Western blot analysis for Wdr4 expression in GNPs infected with lentiviruses carrying Wdr4 WT or mutants at DIV 0 and harvested at DIV 3, showing comparable expression levels among WT and mutants. **M** Western blot analysis for Arhgap17 expression in N2a cells overexpressing Wdr4 WT or mutants. The western blot results were quantified using ImageJ software. The protein levels were normalized first to the Gapdh protein level in each group and then to the corresponding control groups, and expressed as fold changes at the bottom. All western blot analyses were done at least twice.

To substantiate the theory that cell cycle exit by Wdr4 ablation is mediated, at least in part, by the Arhgap17-Rac1 axis, Arhgap17 knockdown and Rac1 activation were used to test their abilities to rescue GNP proliferation defects caused by *Wdr4* deletion. Indeed, *Arhgap17* knockdown in GNPs isolated from P7 *Wdr4 A-cKO* mice significantly increased their proliferation and their tendency to retain cell cycle faculties at DIV3 (detected by Ki67^+^EdU^+^/EdU^+^ signals) (Fig. 7A–C). Similar results were obtained by treating GNPs derived from P7 *Wdr4 A-cKO* mice with a Rac1 activator ML099 [43] (Fig. 7D–F). To test the effect of Rac1 activation in vivo, P3 *Wdr4 A-cKO* and control mice were subcutaneously injected over the cerebellum with ML099 or DMSO, and then harvested at P7. While a reduced number of Ki67^+^ GNPs was observed in the EGL of DMSO-treated *Wdr4 A-cKO* mice compared with control mice, ML099 treatment partially rescued the Ki67^+^ proliferating GNPs in EGL (Fig. 7G, H and S8). These results suggest that Wdr4 promotes cerebellar GNP proliferation by increasing Arhgap17 degradation to facilitate Rac1 activation.

### The disease-associated Wdr4 variants fail to support GNP proliferation

Various *WDR4* mutations were identified in patients with neurodevelopmental disorders, including primordial dwarfism [8–10] and Galloway-Mowat syndrome [11]. In addition to deletion and splicing site mutations, two single residue mutations in human WDR4, D164 and R170 (D166 and R172 in mouse Wdr4) have been reported [8–10]. The mutated residues are conserved and located in the WD domain [8–10], which serves as a scaffold for protein interactions, such as the assembly of a E3 ligase complex and the binding of various substrates [12]. To assess the potential pathogenicity of the mutations, we investigated their abilities to rescue the proliferation defect resulted from Wdr4 deletion by re-expressing WT and mutant Wdr4 in P7 *Wdr4 A-cKO* GNPs. Whereas re-expression of WT Wdr4 increased cell numbers (~40%) and decreased the population of GNPs that exited the cell cycle (~9%), the D166A and R172Q mutants with comparable expression levels failed to rescue these defects in P7 *Wdr4 A-cKO* GNP cultures at DIV3 (Fig. 7I–L). In addition, Arhgap17 protein levels were decreased by the overexpression of WT Wdr4, but not the D166A and R172Q mutants in N2a cells (Fig. 7M). These data provide evidence that the disease-associated Wdr4 mutants are defective in cerebellar GNP proliferation and support their pathogenicity.

## Discussion

Here, we report a novel mechanism by which Wdr4 impacts mouse cerebellar development and locomotion. We show that Wdr4 promotes the ubiquitination and degradation of Arhgap17, thereby facilitating Rac1 activation and preventing the precocious exit of GNPs from the cell cycle. Through this mechanism, Wdr4 promotes GNP proliferation during cerebellum development in a cell-autonomous manner, thereby supporting the expansion of the EGL and IGL. In addition, Wdr4 maintains the organization of Purkinje neurons and the size of the ML in a non-cell-autonomous manner. These functions of Wdr4 in cerebellar development are important for the normal locomotion of animals. Our study suggests that defects in Wdr4-Arhgap17-Rac1 signaling may underlie some symptoms of human cerebellar developmental disorders.

Developmental disorders affect 2–5% of individuals worldwide [44, 45]. However, their heterogeneous causes and varying phenotypes make it difficult to analyze the underlying mechanisms, thus limiting treatment options. Recently, whole-exome sequencing (WES) technology has made progress in linking genetic variants to clinical phenotypes. Through WES, various *WDR4* mutations were identified in patients with neurodevelopmental disorders [8–11]. In addition to growth retardation, global developmental delay, microcephaly, and intellectual disability, the phenotypes of cerebellar atrophy and motor development delay are shared in some of these patients. Importantly, these phenotypes are recapitulated in our *Wdr4* mouse model where *Wdr4* is conditionally deleted in cerebellar GNPs. The consistent phenotypes between the *WDR4*-mutated patients and the *Wdr4*-deficient mouse model suggest a cause-effect relationship, more than an association. In addition, while patients with either homozygous variants, or compound heterozygous variants have neurodevelopmental defects, their parents who carry one WT *WDR4* allele did not display these phenotypes, suggesting that one allele of WT *WDR4* is sufficient to maintain proper function. Importantly, this phenomenon is also recapitulated in the *Wdr4* conditional deletion mouse model. Thus, our mouse model is suitable for studying the underlying mechanisms of cerebellar atrophy caused by *Wdr4* variants and for testing potential therapeutic strategies for these patients.

The Rac1 small GTPase controls various cellular processes, such as proliferation, migration, and differentiation, via a large number of downstream effectors [46]. For instance, Rac1 promotes cell cycle progression by increasing the activity of mTORC [34, 47] and by activating the transcription of *CCND2* (encoding cyclin D2) to facilitate G1/S transition [48]. Consequently, conditional knockout of *Rac1* using a variety of tissue-specific Cre in the nervous system results in accelerated cell cycle exit [40–42], reduced proliferation [40–42, 49], and premature differentiation [42]. Consistently, patients with *RAC1* mutations display neurodevelopmental defects, such as microcephaly, cerebellar atrophy, and intellectual disability [50]. Together, these lines of evidence support our finding that Rac1 is a downstream effector of Wdr4-Arhgap17 signaling, promoting GNP proliferation and cerebellar development through the inhibition of cell cycle exit.

The Shh/Gli pathway is one of the most important pathways to control GNP proliferation, and thus cerebellar development as well as medulloblastoma formation [51, 52]. Even though we did not find out any effector of the Shh/Gli pathway from our proteomic analysis using *Wdr4 A-cKO* GNPs, we do not exclude the possibility that Wdr4 could impact on the Shh/Gli pathway to control GNP proliferation. Indeed, it was shown that Gli1/2 nuclear translocation in response to Shh requires Rac1 activation, and Rac1 is involved in the progression of Shh-type medulloblastoma [38]. Since we found that Wdr4 can regulate Rac1 activity (Fig. 6L), it is possible that Wdr4 regulates the Shh/Gli signaling through Rac1 activation, and therefore promotes GNP proliferation.

We showed that the *Wdr4 A-cKO* mice display a reduction in size of the ML (Fig. 4A, D), a region that is composed of the axons of GNPs and the dendritic arbors of Purkinje neurons [2]. The smaller ML is likely a result of fewer GNPs, which leads to a reduction of GNP axons. However, we do not exclude the possibility that Wdr4 additionally regulates other unidentified proteins that control the formation of synaptic connections between GNP axons and Purkinje dendrites, thereby affecting the expansion of Purkinje dendritic arbors and ML size [53]. Of note, previous studies identified that Wdr4 exerts multiple functions, such as regulating tumor microenvironments [12], stabilizing tRNA [15–17], and maintaining genome stability [18], through interacting with different partners/effectors, including DDB1/PML, METTL1, and FEN1, individually. Therefore, it is possible that Wdr4 acts through additional molecular mechanisms to control cerebellar development, besides Wdr4-Arhgap17-Rac1 signaling. In line with this idea, Rac1 activation only partially rescues the proliferation defect caused by Wdr4 deletion (Fig. 7D–H), implying the existence of other mechanism(s) for mediating the effect of Wdr4 on GNP proliferation. Future studies will be conducted to test this possibility.

In addition to promoting GNP proliferation, several lines of evidence suggest that WDR4 may have functions in other types of cells in the nervous system. First, *Wdr4* is expressed in many cell types in a variety of regions, including in the cortex and cerebellum [32]. Second, the *Wdr4*^*−/−*^ mice have an abnormal brain morphology at E10.5 [18], a stage when neuroepithelial cells are expanded, suggesting that Wdr4 may have a role in regulating neuroepithelial lineage cells and brain formation. Third, patients carrying *Wdr4* variants exhibit brain phenotypes other than cerebellar atrophy and locomotion defects [8–11]. Future studies will be aimed at determining the full range of functions of Wdr4 in different nervous cell types, and their respective underlying mechanisms.

## Supplementary information

Supplementary Figures

Supplementary File - Original Western Blots

Supplementary Table S1

Reproducibility Checklist

Video S1. Nervous system-specific knockout of Wdr4 impairs locomotion (Related to Figure 1)

Video S2A. Wdr4 ablation in cerebellar GNPs impairs locomotion (Related to Figure 5)

Video S2B. Wdr4 ablation in cerebellar GNPs impairs locomotion (Related to Figure 5)

## Data Availability

The proteome data from mouse cerebellar GNPs were deposited to the ProteomeXchange Consortium via the PRIDE partner repository with the dataset identifier PXD022319, which can be accessed via this link: website: http://www.ebi.ac.uk/pride; username: reviewer_pxd022319@ebi.ac.uk; password: HrNpBBRW.

## Materials availability

Materials published in this paper will be available if requested from the corresponding authors.
