# Supplementary material for: Wdr4 promotes cerebellar development and locomotion through Arhgap17-mediated Rac1 activation
Source: Cell Death Dis. 2023 Jan 21;14(1):52. doi: 10.1038/s41419-022-05442-z (PMC9867761; doi:10.1038/s41419-022-05442-z)
Supplement: Supplementary file 1 — Supplementary Figures [file 41419_2022_5442_MOESM1_ESM.pdf]

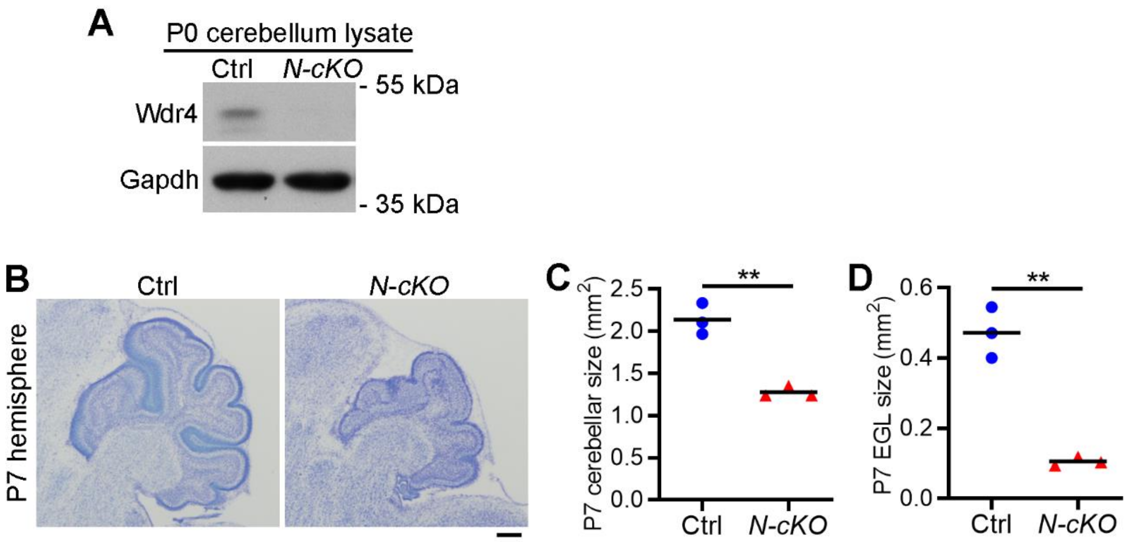

Figure S1. Nervous system-specific *Wdr4* deletion impairs cerebellum development (Related to Figure 1)

(A) Western blot analysis for Wdr4 expression in the P0 *Wdr4 N-cKO* and control cerebella. The western blot analysis was done at least twice.

(B-D) Representative Nissl staining images (B) and quantitative data (C, D) for cerebellar size (B, C), foliation (B), and EGL (B, D) in the P7 *Wdr4 N-cKO* and control cerebellar hemispheres. Scale bar, 250 μm. Data were from 3 cerebella in each group and analyzed using two-tailed unpaired Student's *t*-test without Welch's correction (equal variances), $p = 0.0017$ in (C), 0.0010 in (D). Data are represented as individual points and mean; $^{**}p < 0.005$.

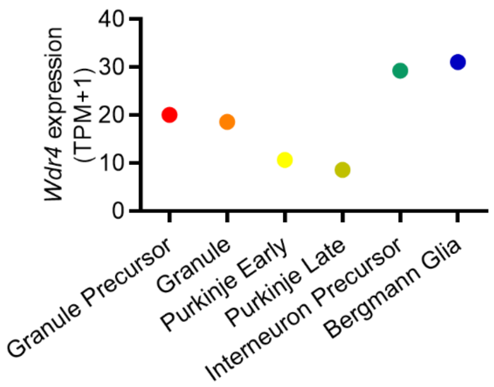

Figure S2. *Wdr4* mRNA expression in various cell types in the developing cerebellum (Related to Figure 2)

Wdr4 mRNA levels in the indicated cell types in the cerebellum as indicated by the single cell RNA-seq data retrieved from a previous study (Rosenberg et al., 2018). Values are shown as Transcripts Per Kilobase Million (TPM) +1.

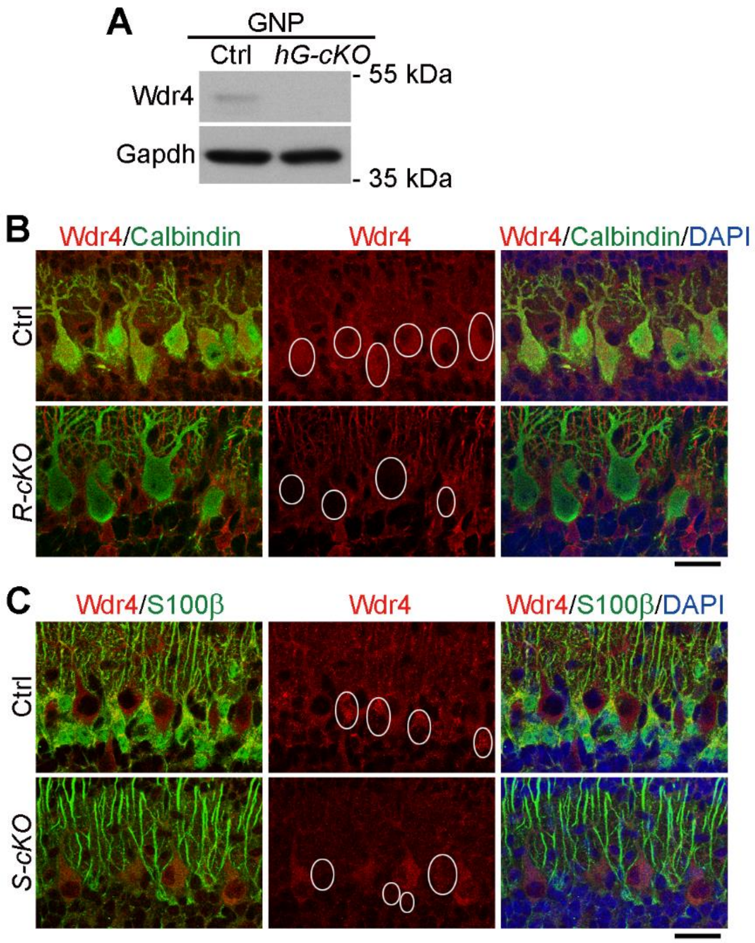

Figure S3. Wdr4 protein expression in various cKO and control mouse cerebella (Related to Figure 2)

(A) Western blot analysis for Wdr4 expression in the P7 *Wdr4 hG-cKO* and control GNPs. The western blot analysis was done at least twice.

(B-C) Representative confocal images showing Wdr4 expression in the P7 *Wdr4 R-cKO* (B), *Wdr4 S-cKO* (C), and control cerebella. White circles mark Wdr4 expression in Calbindin^{+} Purkinje neurons (B), and in S100β^{+} Bergmann glia (C). Wdr4 was depleted in Calbindin^{+} Purkinje neurons in *Wdr4 R-cKO* (B, lower panels), and in S100β^{+} Bergmann glia in *Wdr4 S-cKO* (C, lower panels). Scale bars, 25 μm.

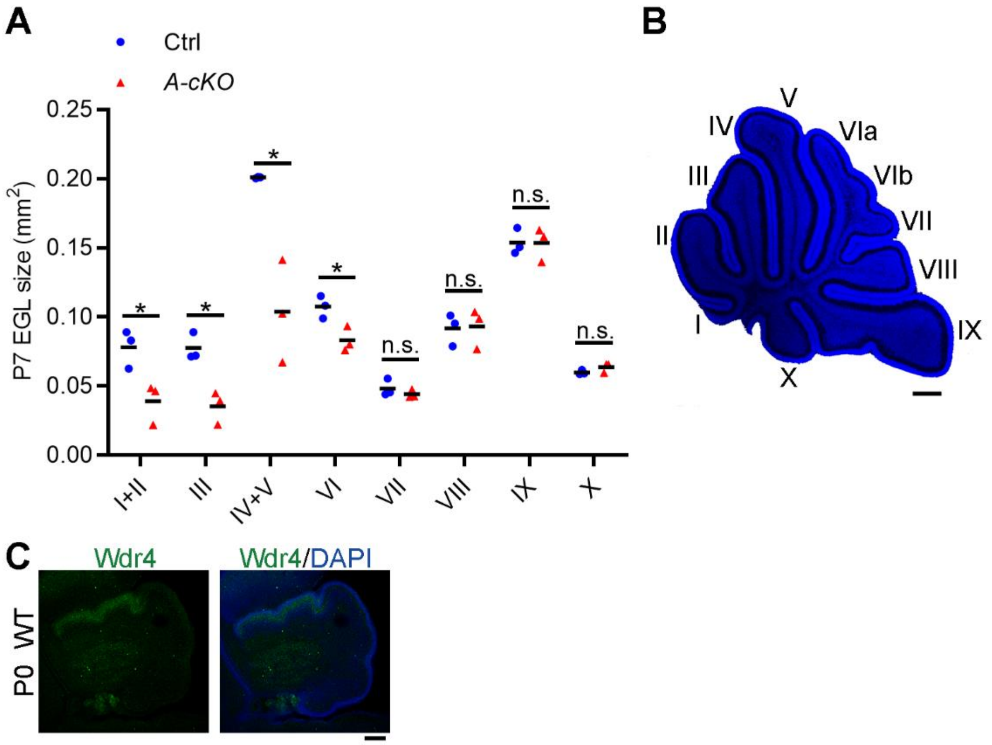

Figure S4. *Wdr4 A-cKO* mice exhibit a reduced EGL size in the anterior and central lobules of cerebellum (Related to Figure 2)

(A) Quantitative data showing the EGL size in the indicated cerebella lobules of the P7 *Wdr4 A-cKO* and control mice. Data were from 3 cerebella in each group and analyzed using two-tailed unpaired Student's *t*-test without Welch's correction (equal variances), p = 0.0283 in lobule I+II, 0.0092 in lobule III, 0.0450 in lobule IV+V, 0.0263 in lobule VI, 0.3575 in lobule VII, 0.9068 in lobule VIII, 0.9794 in lobule IX, and 0.1540 in lobule X. Data are represented as individual points and mean; *p < 0.05; n.s., non-significant.

(B) A representative confocal image of a P7 WT cerebellum showing the 10 different lobules quantified. Scale bar, 250 μm.

(C) Representative confocal images of a P0 WT cerebellum showing Wdr4 protein expression in the EGL of lobule I-VI. Scale bar, 150 μm.

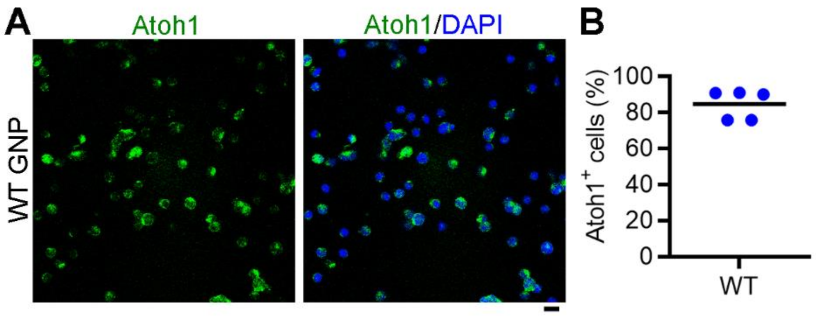

Figure S5. Most of the cells in the GNP cultures express the GNP-specific marker Atoh1 (Related to Figure 3)

(A-B) Representative confocal images (A) and quantitative data (B) showing that ~85% of cells isolated from P7 mouse cerebella are Atoh1^{+} GNPs. The cells were isolated using Percoll density gradient, and then cultured for 3 days before staining. Scale bar, 25 μm. Data were from 5 repeats, and are represented as individual points and mean.

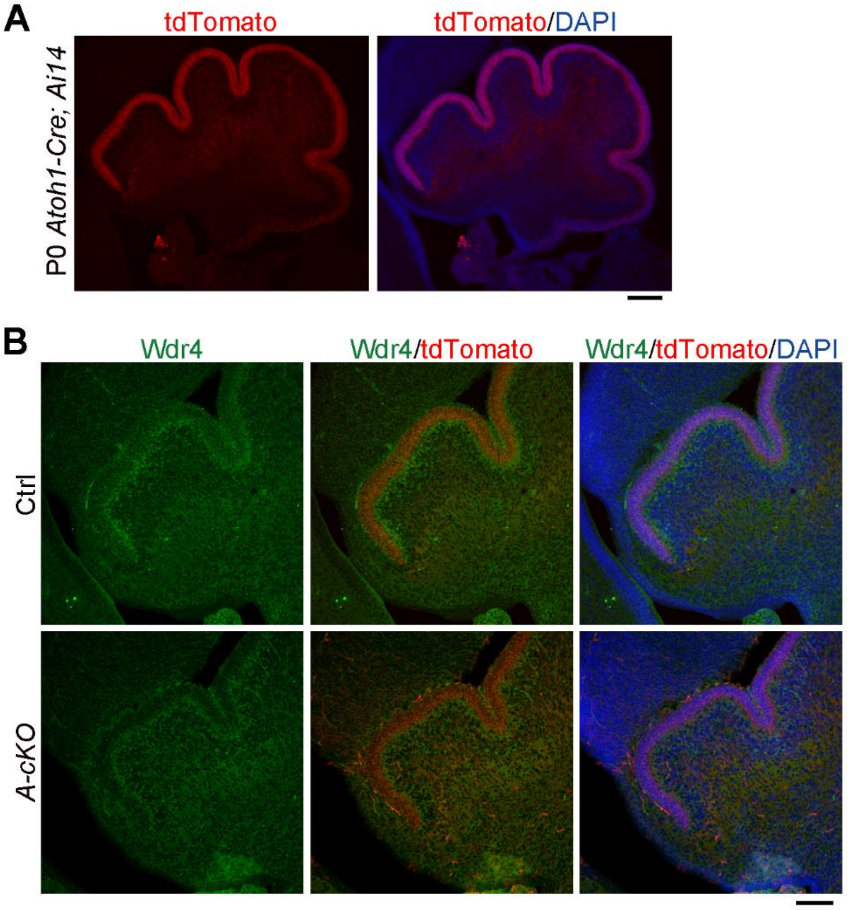

Figure S6. Wdr4 protein expression in *Wdr4 A-cKO* and control mouse cerebella (Related to Figure 3)

(A) Representative confocal images of the P0 *Atoh1-Cre; Ai14*$^{flox/+}$ cerebellum showing the tdTomato signal in the EGL of every lobule. Scale bar, 150 μm.

(B) Representative confocal images of the P0 *Wdr4 A-cKO; Ai14* and control cerebella showing the deletion of Wdr4 in the EGL of *Wdr4 A-cKO* mice. Scale bar, 100 μm.

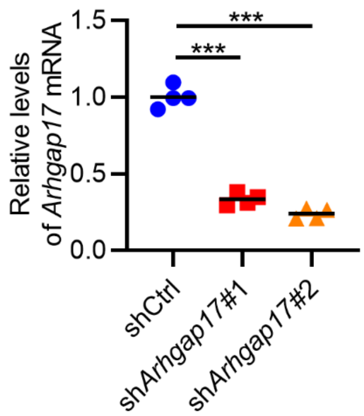

Figure S7. The levels of *Arhgap17* mRNA are reduced by *Arhgap17* shRNAs (Related to Figure 7)

RT-qPCR analysis for the expression of *Arhgap17* mRNA in N2a cells expressing control or *Arhgap17* shRNAs. Data were from 4 repeats in each group and analyzed using one-way ANOVA post hoc Dunnett's test, $p < 0.0001$ in both sh*Wdr4*#1 v.s. shCtrl, and sh*Wdr4*#2 v.s. shCtrl. Data are represented as individual points and mean; ***$p < 0.0005$.

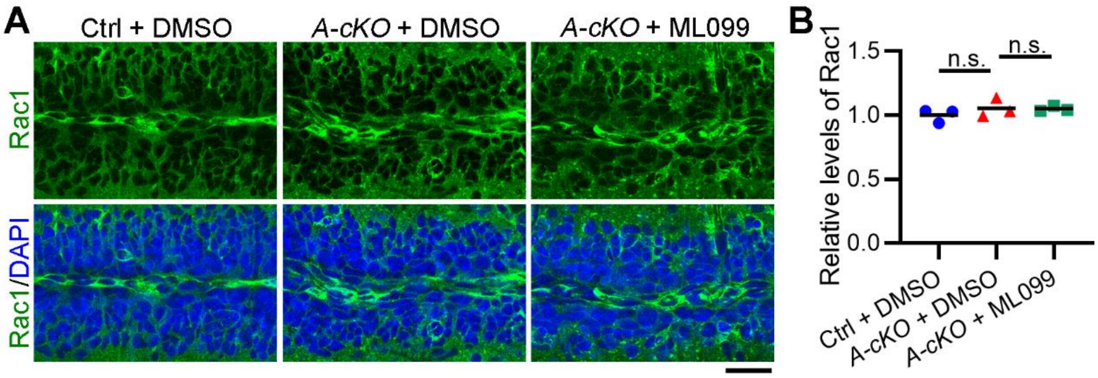

Figure S8. Rac1 expression in the *Wdr4 A-cKO* and control cerebella (Related to Figure 7)

(A-B) Representative confocal images (A) and quantitative data (B) showing Rac1 expression in the GNPs in the P7 *Wdr4 A-cKO* and control cerebella treated with DMSO or ML099. Scale bar, 25 µm. Data were from 3 cerebella in each group and analyzed using one-way ANOVA post hoc Turkey's test, p = 0.4854 in (B, *A-cKO* v.s. Ctrl with DMSO), and = 0.9960 in (B, ML099 v.s. DMSO in *A-cKO*). Data are represented as individual points and mean; n.s., non-significant.

Video S1. Nervous system-specific knockout of *Wdr4* impairs locomotion (Related to Figure 1)

A representative video showing the ataxic gait of the P14 *Wdr4 N-cKO* mouse compared to the control.

Video S2. *Wdr4* ablation in cerebellar GNPs impairs locomotion (Related to Figure 5)

(A and B) Representative videos showing the impaired gait phenotypes in the 2-month-old *Wdr4 A-cKO* mouse (A), including duck feet, an upright tail, and a slower walking speed, compared to the control (B).
