# Supplementary material for: Wdr4 promotes cerebellar development and locomotion through Arhgap17-mediated Rac1 activation
Source: Cell Death Dis. 2023 Jan 21;14(1):52. doi: 10.1038/s41419-022-05442-z (PMC9867761; doi:10.1038/s41419-022-05442-z)

Uncropped western blots

Figure 6B

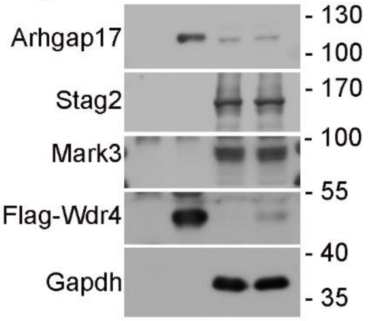

Figure 6C

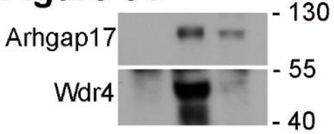

Figure 6D

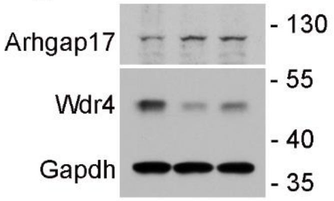

Figure 6F

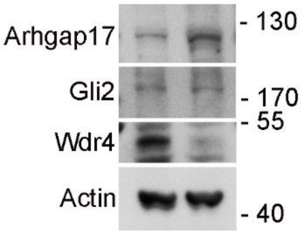

Figure 6G

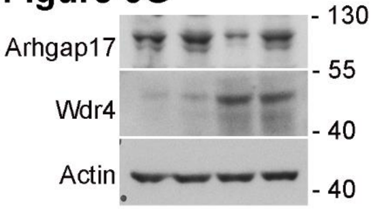

Figure 6H

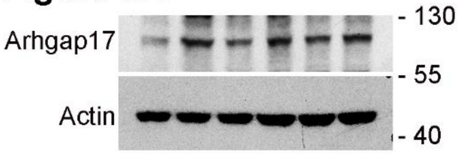

Figure 6I

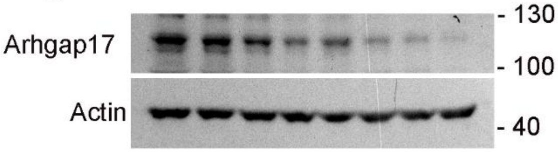

Figure 6J

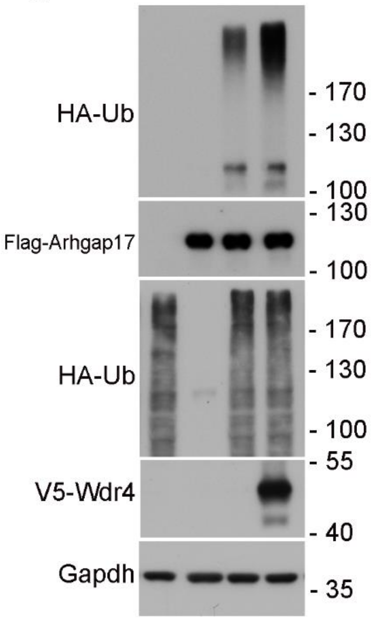

Figure 6K

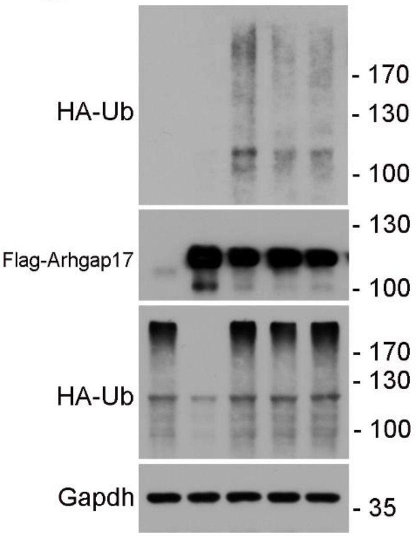

Figure 6L

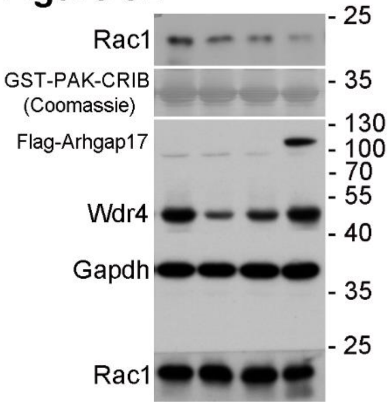

Figure 7L

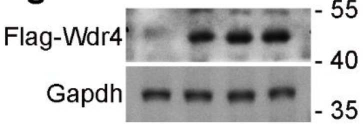

Figure 7M

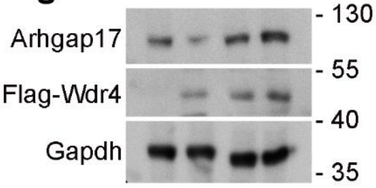

Figure S1A

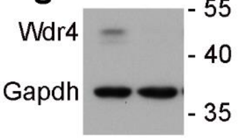

Figure S3A

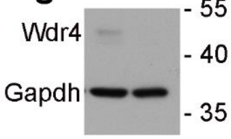

Supplement: Supplementary file 2 — Supplementary File - Original Western Blots [file 41419_2022_5442_MOESM2_ESM.pdf]
